# Selective endocytosis of recombinant human BMPs through cell surface heparan sulfate proteoglycans in CHO cells: BMP-2 and BMP-7

**DOI:** 10.1038/s41598-021-82955-1

**Published:** 2021-02-09

**Authors:** Mi Gyeom Kim, Che Lin Kim, Young Sik Kim, Ju Woong Jang, Gyun Min Lee

**Affiliations:** 1 Department of Biological Sciences, KAIST, 291 Daehak-ro, Yuseong-gu, Daejeon, 34141 Republic of Korea; 2 The Novo Nordisk Foundation Center for Biosustainability, Technical University of Denmark, Lyngby, Denmark; 3 Institute of Biomaterial and Medical Engineering, Cellumed, Seoul, Republic of Korea

**Keywords:** Biological techniques, Biotechnology

## Abstract

Cell surface heparan sulfate proteoglycan (HSPG)-mediated endocytosis results in poor yields of recombinant human bone morphogenetic proteins (rhBMPs) from CHO cell cultures. Upon incubation of rhBMP-2 and rhBMP-7 with CHO cells at 37 °C, both rhBMP-2 and rhBMP-7 bound to the cell surface HSPGs in CHO cells, but only rhBMP-2 was actively internalized into CHO cells. Cell surface HSPGs were found to serve as the main receptor for rhBMP-2 internalization. It was also found that the cell surface HSPG-mediated endocytosis of rhBMP-2 occurred through both the clathrin- and caveolin-dependent pathways. Blockage of rhBMP-2 internalization by the addition of structural analogs of HSPGs such as dextran sulfate (DS) and heparin dramatically increased rhBMP-2 production in recombinant CHO (rCHO) cell cultures. Compared to the control cultures, addition of DS (1.0 g/L) and heparin (0.2 g/L) resulted in a 22.0- and 19.0-fold increase in the maximum rhBMP-2 concentration, respectively. In contrast, the production of rhBMP-7, which was not internalized into the rCHO cells, did not dramatically increase upon addition of DS and heparin. Taken together, rhBMPs have a different fate in terms of HSPG-mediated internalization in CHO cells. HSPG-mediated endocytosis of each rhBMP should be understood individually to increase the rhBMP yield in rCHO cell cultures.

## Introduction

Bone morphogenetic proteins (BMPs) are a group of multifunctional cytokines involved in bone and cartilage regeneration^1^. Based on the amino acid homology at the C-terminus and their ability to bind certain type I receptors, members of the BMP family are generally classified into four categories: BMP-2/4, BMP-5/6/7/8a/8b, BMP-9/10, and BMP-12/13/14^2^. Among them, recombinant human BMP-2 (rhBMP-2) and rhBMP-7 produced in CHO cells have been clinically used to treat non-unions, long bone fractures, and spinal infusion^3^. However, despite significant efforts to increase the product yields^4–6^ achieving high titers of functionally active rhBMP-2 and rhBMP-7 in CHO cell cultures still remains challenging.

Since endocytic regulation plays a critical role in modulating the extracellular level of BMPs^7^, secreted rhBMPs in the culture medium may be the targets of internalization and endosomal degradation during cell culture. In fact, endocytosis of secreted rhBMP-4 through cell surface heparan sulfate proteoglycans (HSPGs), which served as the major receptors, results in poor yields of rhBMP-4 from CHO cell cultures^8^. The heparan sulfate (HS) chain of HSPGs is a linear polysaccharide that provides docking sites for the HS-binding ligands via electrostatic interactions^9^. Addition of dextran sulfate (DS), a competitive inhibitor of HSPGs, to the culture medium significantly increases rhBMP-4 production through the blockage of rhBMP-4 internalization in CHO cell cultures^8^. Previously, DS was also added to the culture medium to increase the rhBMP-2 production, but the mechanism by which DS mediates enhanced rhBMP-2 production has not been understood yet^10^. In fact, it has never been examined before whether endocytosis of secreted rhBMP-2 and rhBMP-7 through cell surface-bound HSPGs occurs during CHO cell cultures.

BMP-2, similar to its closely related paralogue BMP-4, has an HS-binding motif at the N-terminus^11,12^. Thus, the mature rhBMP-2 dimer has a total of two HS-binding domains, each located on the opposite side of the dimer fingers. In contrast, based on structure predictions and mapping of electrostatic surface, BMP-7 is predicted to have an HS-binding motif at the C-terminus^11–13^. It is also predicted that the mature rhBMP-7 dimer, unlike the mature rhBMP-2 dimer, has a single HS-binding domain that originates from two C-terminal motifs arranged in proximity within the wrist/palm region^11^. The functional roles and HS binding may differ between rhBMP-2 and rhBMP-7 due to structural differences.

In this study, we investigated the endocytosis of secreted rhBMP-2 and rhBMP-7 into CHO cells in an effort to obtain high yields of rhBMP-2 and rhBMP-7, respectively. We found that only rhBMP-2 was internalized into CHO cells via cell surface HSPG binding. Based on this finding, we used DS and heparin to enhance the rhBMP-2 yield through blockage of rhBMP-2 internalization.

## Results

### Different fates of extracellular rhBMP-2 and rhBMP-7 in DG44 cells

To study whether extracellular rhBMP-2 and rhBMP-7 are internalized via cell surface HSPGs into CHO cells, medium containing 10 μg/mL of rhBMP-2 or rhBMP-7 was incubated at 4 °C and 37 °C with and without DG44 cells in the absence or presence of 0.5 g/L DS or 0.1 g/L heparin. At 4 °C, the rhBMPs cannot be actively internalized into the cells^15^.

**Figure 1:**
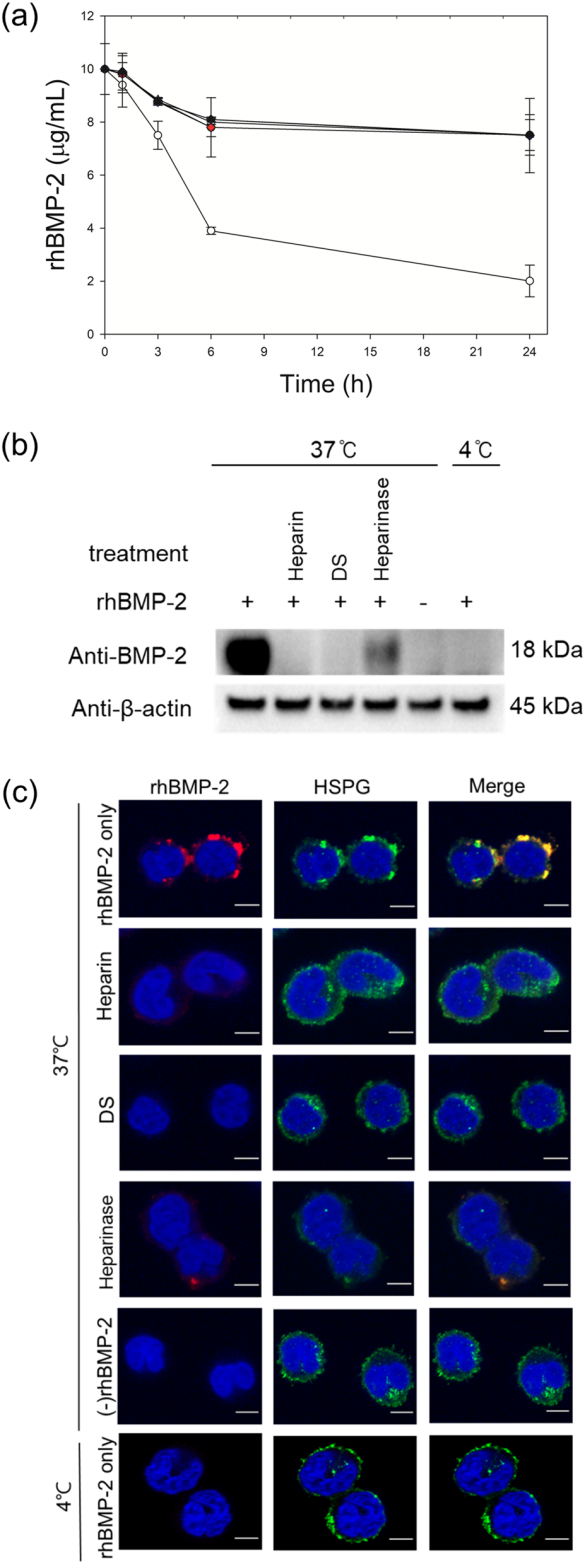
Active internalization of rhBMP-2 into DG44 cells. (**a**) Profiles of rhBMP-2 during incubation with and without cells. With cells at 37 °C in the absence of heparin and DS (white circle), with cells at 37 °C in the presence of 0.1 g/L heparin (blue diamond) or 0.5 g/L DS (red diamond), with cells at 4 °C in the absence of heparin and DS (green circle), without cells in the absence of heparin and DS at 37 °C (white triangle) or 4 °C (black triangle). The error bars represent standard deviations calculated from six independent experiments. (**b**) Western blot analysis of intracellular rhBMP-2 at 37 °C or 4 °C. ‘+’ and ‘−’ indicate presence and absence, respectively. ‘Heparinase’ indicates cells pretreated with heparinase I/III. β actin was used as the loading control. (**c**) Confocal microscopy to examine the intracellular location of rhBMP-2. Intracellular rhBMP-2 was stained red, while HSPGs were stained green. The nuclei were stained blue using DAPI. The merge images indicate co-localization (yellow) of rhBMP-2 and HSPGs. Scale bar 5 μm.

As shown in Fig. 1a, after 24 h, the rhBMP-2 concentration in the medium without DG44 cells decreased to approximately 7.5 μg/mL at both the temperatures. The decrease in rhBMP-2 concentration in the medium was also confirmed using western blot analysis (Supplementary Fig. S1). This instability of rhBMP-2 in the medium is in accordance with previous studies in which rhBMP-2 has been shown to precipitate and undergo a conformational change at neutral pH^16,17^.

In DG44 cells, the rhBMP-2 concentration significantly decreased to 2.5 ± 0.6 μg/mL after 24 h at 37 °C (*p* < 0.01, n = 6), whereas it decreased to 7.5 ± 0.8 μg/mL after 24 h at 4 °C. When DS or heparin was added to the medium, the rhBMP-2 concentration after 24 h at 37 °C was similar to that after 24 h at 4 °C (Fig. 1a). This result suggests that extracellular rhBMP-2 was actively internalized through surface HSPGs into DG44 cells.

To confirm the internalization of rhBMP-2, cells incubated with rhBMP-2 for 3 h at 4 °C and 37 °C were harvested after removing the surface-bound rhBMP-2 with an acidic buffer. Intracellular rhBMP-2 was barely detected in the lysate of cells at 4 °C, but was detected in the lysate of cells at 37 °C (Fig. 1b). Intracellular rhBMP-2 was barely detected in the lysate of cells incubated with DS or heparin at 37 °C, confirming that rhBMP-2 was actively internalized through HSPGs into DG44 cells. In addition, western blot analysis of cells treated with heparinase also confirmed this hypothesis. Treatment of cells with heparinase I/III reduced the cellular uptake of rhBMP-2 by 72.7 ± 5.5% (*p* < 0.01, n = 5; Fig. 1b). Furthermore, using immunofluorescence confocal microscopy, rhBMP-2 (red) was found to co-locate intracellularly with HSPGs (green) in cells without DS or heparin at 37 °C, but not in cells with DS or heparin at 37 °C (Fig. 1c). Confocal microscopy also showed that treatment of cells with heparinase I/III removed a significant portion of the cell surface HSPGs, resulting in decreased cellular uptake of rhBMP-2 (Fig. 1c).

**Figure 2:**
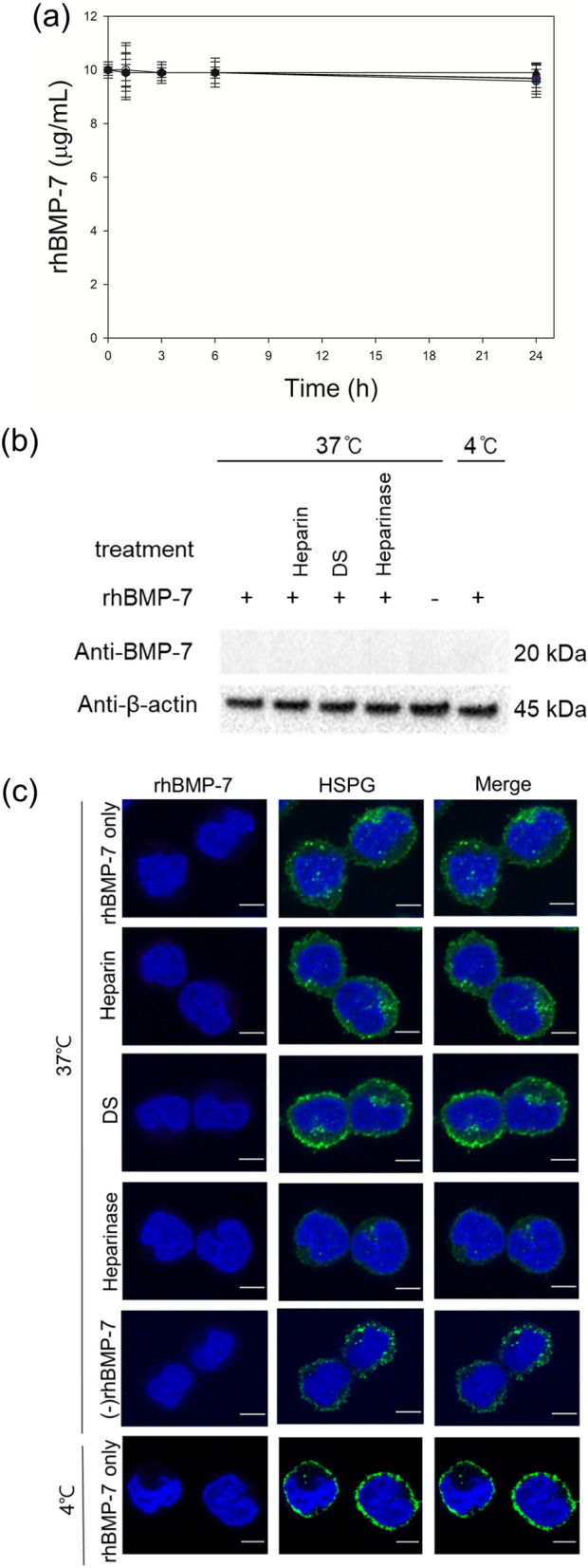
No internalization of rhBMP-7 in DG44 cells. (**a**) Profiles of rhBMP-7 during incubation with and without cells. With cells at 37 °C in the absence of heparin and DS (white circle), with cells at 37 °C in the presence of 0.1 g/L heparin (blue diamond) or 0.5 g/L DS (red diamond), with cells at 4 °C in the absence of heparin and DS (green circle), without cells in the absence of heparin and DS at 37 °C (white triangle) or 4 °C (black triangle). The error bars represent standard deviations calculated from six independent experiments. (**b**) Western blot analysis of intracellular rhBMP-7 at 37 °C or 4 °C. ‘ + ’ and ‘−’ indicate presence and absence, respectively. ‘Heparinase’ indicates cells pretreated with heparinase I/III. β actin was used as the loading control. (**c**) Confocal microscopy to examine the intracellular location of rhBMP-7. Intracellular rhBMP-7 was stained red, while HSPGs were stained green. The nuclei were stained blue. The merge images indicate co-localization (yellow) of rhBMP-7 and HSPGs. Scale bar 5 μm.

Unlike rhBMP-2, the rhBMP-7 concentration in the medium remained almost constant after 24 h at both the temperatures, regardless of the presence of DG44 cells and competitive inhibitors of HSPGs (*p* ≥ 0.05, n = 6; Fig. 2a and Supplementary Fig. S2). Furthermore, intracellular rhBMP-7 was not detected using western blot analysis (Fig. 2b) and confocal microscopy (Fig. 2c). Thus, rhBMP-7 is not internalized into DG44 cells.

### Binding of rhBMP-2 and rhBMP-7 to cell surface HSPGs in DG44 cells

To understand the different fates of extracellular rhBMP-2 and rhBMP-7, the interaction between rhBMPs and cell surface HSPGs in DG44 cells was examined using confocal microscopy. Cells were incubated at 4 °C for 1 h with 10 μg/mL rhBMP-2 or rhBMP-7, followed by antibody staining.

**Figure 3:**
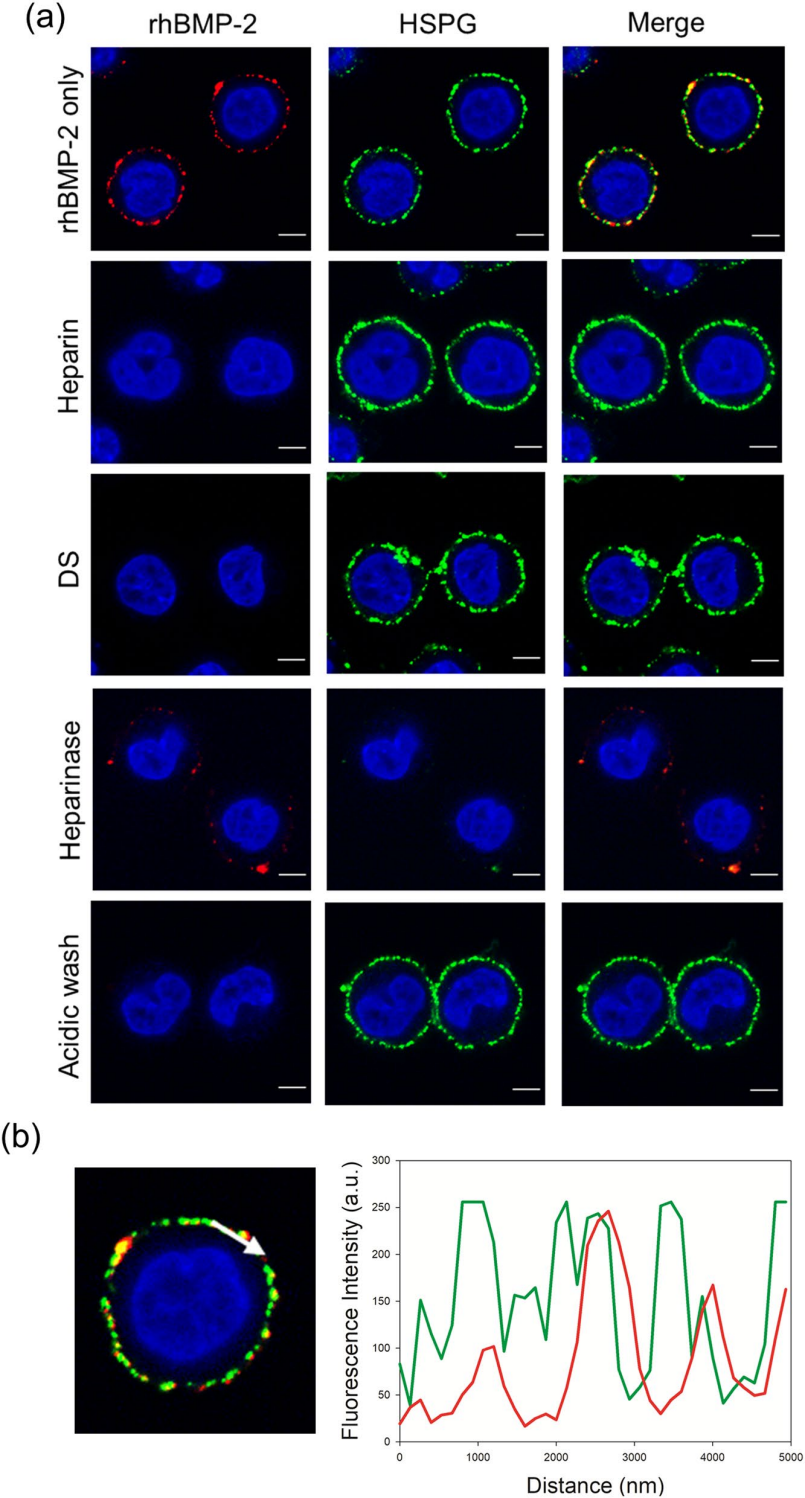
Binding of rhBMP-2 to cell surface HSPGs in DG44 cells. (**a**) Confocal microscopy to examine rhBMP-2 binding to cell surface HSPGs in the absence or presence of heparin and DS. Cells were incubated for 1 h at 4 °C with purified rhBMP-2 (10 μg/mL) alone or in the presence of heparin (0.1 g/L) or DS (0.5 g/L). Cells pre-treated with heparinase I/III (indicated as heparinase) and cells washed with acidic buffer (indicated as acid buffer) were also incubated for 1 h at 4 °C with purified rhBMP-2 alone. rhBMP-2 was stained red, while HSPGs were stained green. Nuclear counterstaining (blue) was carried out using DAPI. The merge image highlights co-localization (yellow) of rhBMP-2 and HSPGs. Scale bar, 5 μm. (**b**) The fluorescence intensity profile of HSPGs (green) and rhBMP-2 (red) across the arrow shown in a confocal image in Fig. 3b.

The merged overlay image of cells incubated with rhBMP-2 revealed co-localization of rhBMP-2 and cell surface HSPGs (Fig. 3a). The fluorescence intensity profile across the arrow for both the green and red channels confirmed the co-localization of rhBMP-2 and cell surface HSPGs (Fig. 3b). Overall, the fluorescence intensity peak of rhBMP-2 followed that of HSPG. In contrast, co-localization of rhBMP-2 and cell surface HSPGs was hardly detected in cells incubated with DS or heparin and in cells washed with acid. Moreover, treatment of cells with heparinase I/III significantly reduced the binding capacity of rhBMP-2 to cell surface HSPGs (Fig. 3a).

**Figure 4:**
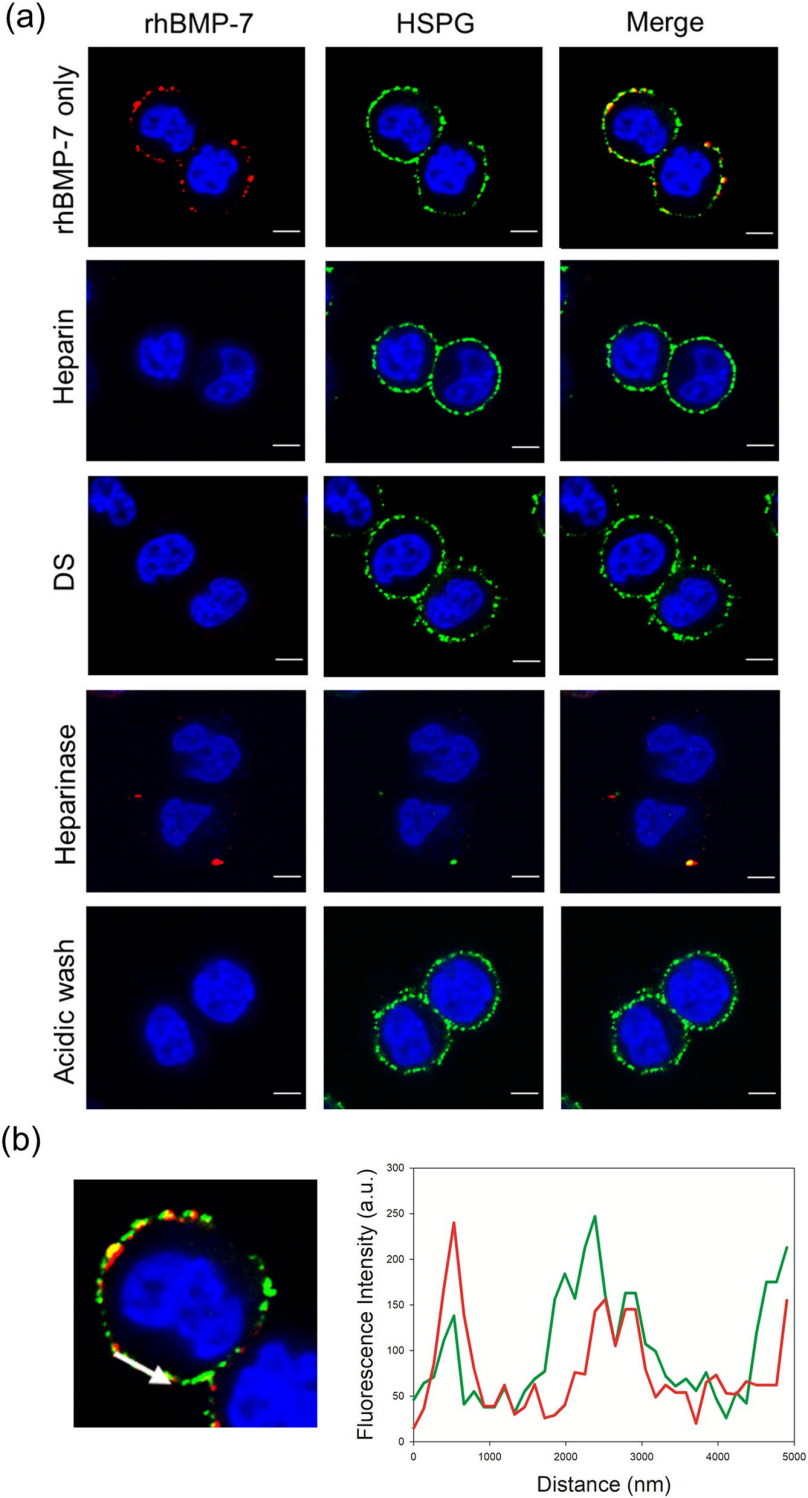
Binding of rhBMP-7 to cell surface HSPGs in DG44 cells. (**a**) Confocal microscopy to examine rhBMP-7 binding to cell surface HSPGs in the absence or presence of heparin and DS. Cells were incubated for 1 h at 4 °C with purified rhBMP-7 (10 μg/mL) alone or in the presence of heparin (0.1 g/L) or DS (0.5 g/L). Cells pre-treated with heparinase I/III (indicated a heparinase) and cells washed with acidic buffer (indicated as acid buffer) were also incubated for 1 h at 4 °C with purified rhBMP-7 alone. rhBMP-7 was stained red, while HSPGs were stained green. Nuclear counterstaining (blue) was carried out using DAPI. The merge image highlights colocalization (yellow) of rhBMP-7 and HSPGs. Scale bar, 5 μm. (**b**) The fluorescence intensity profile of HSPGs (green) and rhBMP-7 (red) across the arrow in a confocal image shown in Fig. 4b.

Similar to rhBMP-2, rhBMP-7 co-localized with cell surface HSPGs in DG44 cells (Fig. 4a,b). In addition, digestion of cell surface HSPGs with heparinase I/III significantly reduced the binding of rhBMP-7 to cell surface HSPGs (Fig. 4a). Thus, both rhBMP-2 and rhBMP-7 bind to cell surface HSPGs in DG44 cells, but only rh-BMP-2 is internalized into DG44 cells.

### Transient knockdown of rhBMP-2-binding molecules in DG44 cells

To investigate the role of cell surface HSPGs in the internalization of rhBMP-2, BMP-binding molecules including HSPGs in DG44 cells were transiently knocked down using targeted siRNAs, followed by rhBMP-2 internalization assay. The target BMP-binding molecules were BMP receptors (*bmpr1a* and *bmpr2*), a BMP antagonist (*twsg1*), and an essential enzyme for HSPG synthesis (*xylt2*), which are highly expressed in DG44 cells^8^.

**Figure 5:**
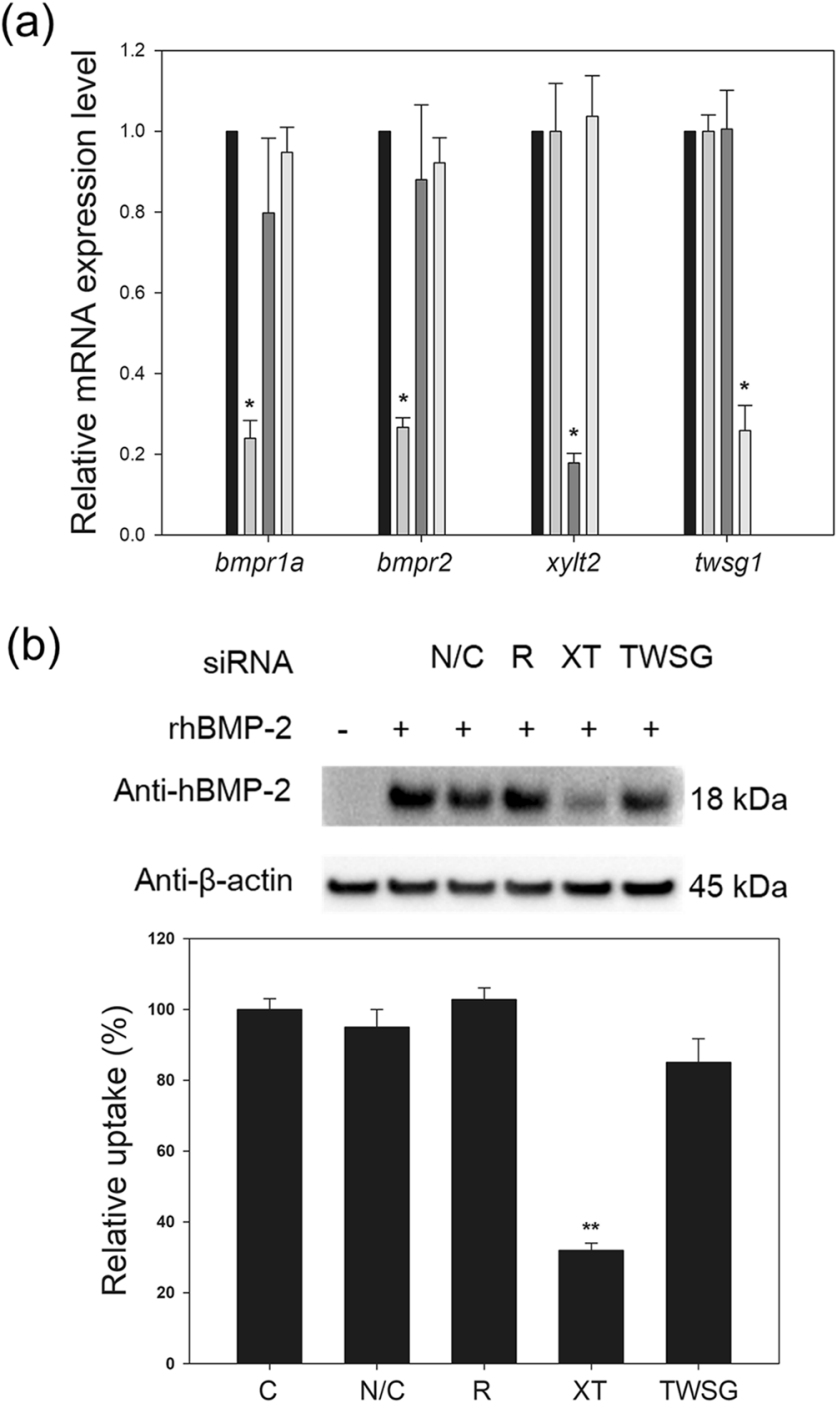
Effects of transient knockdown of rhBMP-2-binding proteins on rhBMP-2 internalization in DG44 cells. (**a**) Relative mRNA expression levels of bmpr1a, bmpr2, xylt2, and twsg1. Cells were transfected with siRNAs targeting both bmpr1a and bmpr2 (light gray), xylt2 (dark gray), or twsg1 (white). Values were normalized to gapdh and then calculated based on the value of the negative control siRNA (N/C)‐transfected cells (black). The error bars indicate standard deviations calculated from the data of three independent experiments. (**b**) Western blot analysis of intracellular rhBMP‐2 in non-transfected cells (C) and cells transiently transfected with N/C, siRNAs targeting bmpr1a and bmpr2 (R), xylt2 (XT), or twsg1 (TWSG) (top). β‐actin was used as the loading control. The band intensity was calculated based on the value of the N/C (bottom). The error bars indicate standard deviations calculated from the data of three independent experiments. *p < 0.05, *p < 0.01.

As shown in Fig. 5a, the mRNA levels of these target genes were reduced by 72%-82%, compared to the levels in cells transfected with a negative control siRNA. However, the rhBMP-2 internalization assay revealed that only the knockdown of HSPG significantly reduced the cellular uptake of rhBMP-2 by approximately 69% (*p* < 0.01, n = 4; Fig. 5b). The knockdown of neither the receptors nor the BMP antagonist significantly affected the cellular uptake of rhBMP-2 (*p* ≥ 0.05), indicating that cell surface HSPGs act as the main receptor in the internalization of rhBMP-2.

### Effect of endocytosis inhibitors on rhBMP-2 uptake in DG44 cells

The pathway of rhBMP-2 endocytosis depends on the cell line^18,19^. To identify the pathway of rhBMP-2 endocytosis in CHO cells, DG44 cells were pretreated with endocytosis inhibitors acting on different pathways (chlorpromazine, genistein, and dynasore). Cells that were not pretreated with endocytosis inhibitors and pretreated with 20 μL DMSO, which was used in the stock solutions of genistein and dynasore, served as controls. The pretreated cells were then incubated for 3 h with IMDM plus 1 × HT containing 10 μg/mL rhBMP-2. Chlorpromazine disturbs clathrin-mediated endocytosis, genistein disrupts caveolin-mediated endocytosis, and dynasore inhibits both clathrin- and caveolin-mediated endocytosis by blocking the GTPase activity of dynamin^20^. The concentration of each inhibitor was determined according to previous studies^8^.

**Figure 6:**
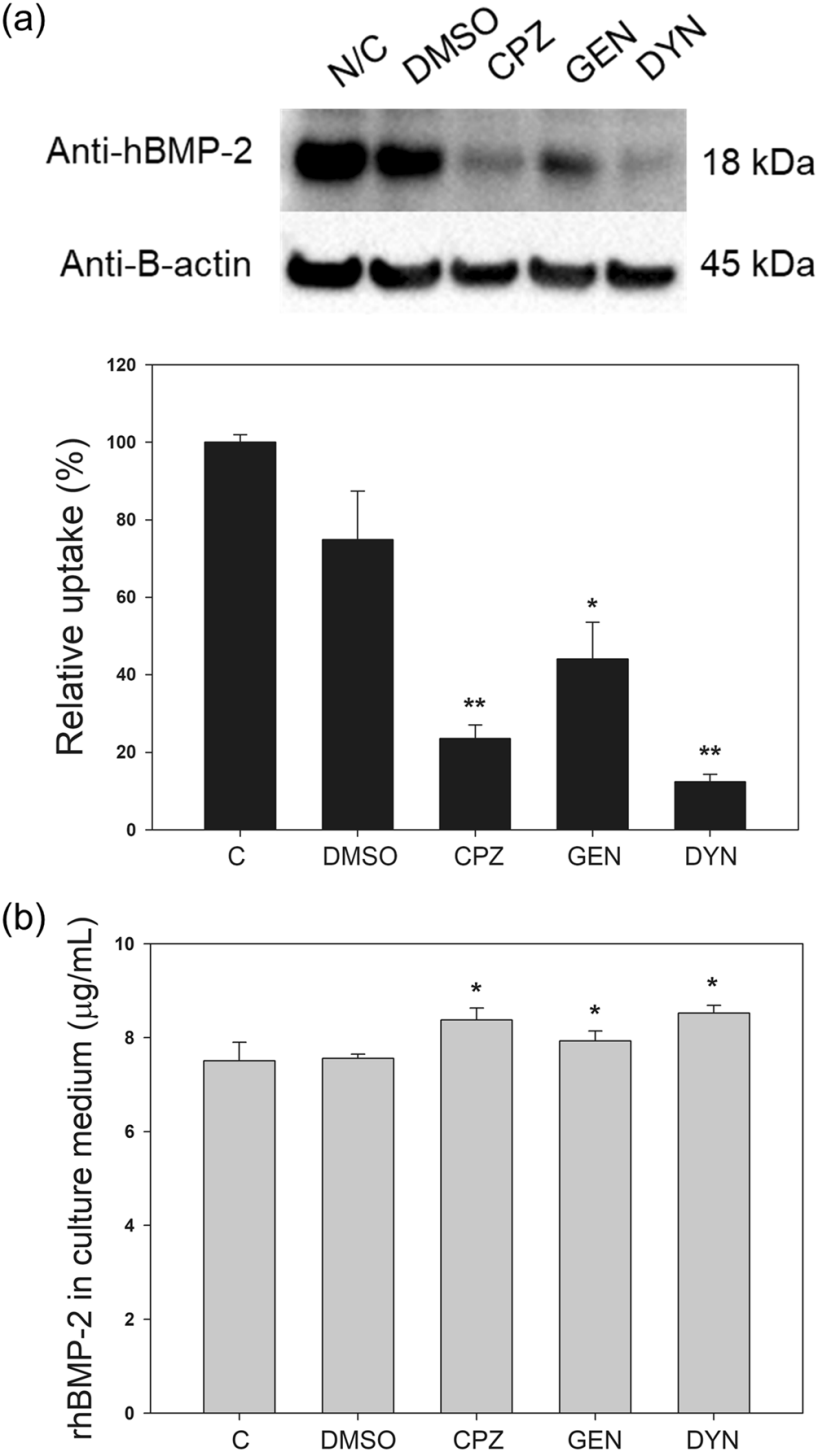
Effects of endocytosis inhibitors on rhBMP-2 internalization in DG44 cells. (**a**) Western blot analysis of intracellular rhBMP‐2 in cells without any treatment as a control (C) and cells pre-incubated with DMSO as a vehicle control, chlorpromazine (CPZ,30 μM), genistein (GEN, 200 μM), or dynasore (DYN, 100 μM) for 30 min before addition of rhBMP-2 (top). β‐actin was used as the loading control. The relative rhBMP-2 uptake (%) was normalized to the rhBMP-2 uptake of C (100%, bottom). (**b**) The rhBMP-2 concentration in the culture medium after 3 h of incubation. The error bars indicate standard deviations calculated from the data of three independent experiments. *p < 0.05, *p < 0.01.

As shown in Fig. 6a, internalization of rhBMP-2 was significantly reduced by all three inhibitors (p < 0.05, n = 3), whereas it was not significantly affected by DMSO alone (p ≥ 0.05, n = 3). In addition, after 3 h of incubation, the rhBMP-2 concentration in the culture medium with cells pretreated with the inhibitor was significantly higher than that of the control (Fig. 6b). These results indicate that both clathrin- and caveolin-mediated endocytic pathways are the routes for rhBMP-2 internalization in DG44 cells. Confocal microscopy also confirmed the intracellular co-localization of rhBMP-2 and HSPGs (pink), rhBMP-2 and clathrin or caveolin (orange), HSPGs and clathrin or caveolin (yellow-green), and rhBMP-2, HSPGs, and clathrin/caveolin (white) in the endocytic punta (Supplementary Fig. S3).

### CHO-BMP-2 and CHO-BMP-7 cell cultures supplemented with DS or heparin

To inhibit HSPG-mediated internalization of rhBMP-2, CHO-BMP-2 cells were cultivated in medium supplemented with DS (0.5 or 1.0 g/L) or heparin (0.1 or 0.2 g/L). The concentrations of DS and heparin used in the culture medium were determined previously^8^. Cell culture without inhibitors was used as a control. For comparison, CHO-BMP-7 cells were cultivated in the same manner.

**Figure 7:**
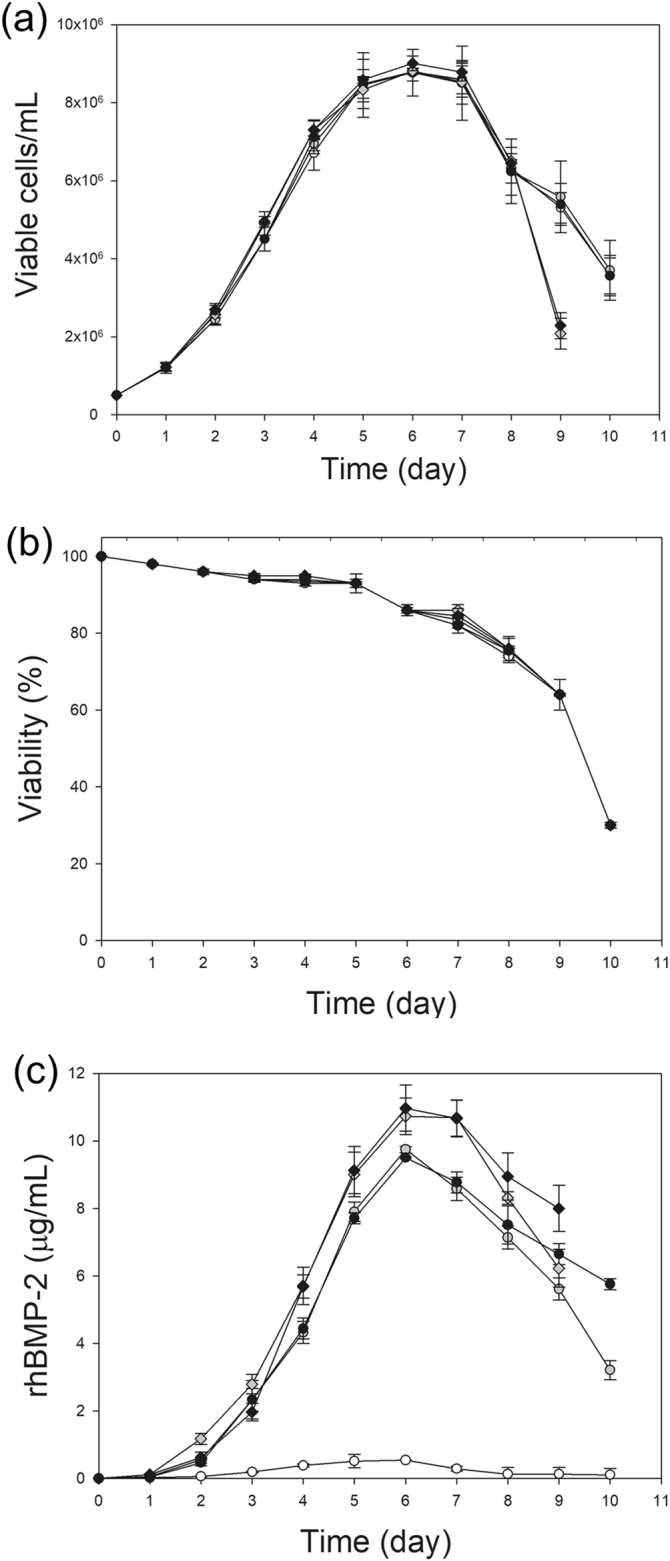
Supplementation of DS or heparin in CHO-BMP-2 cells cultures. Profiles of (**a**) cell growth, (**b**) viability, and (**c**) rhBMP-2 concentration during cultures supplemented with DS or heparin. No supplementation (white circle), supplementation with 0.1 g/L heparin (gray square), 0.2 g/L heparin (black square), 0.5 g/L DS (gray diamond), and 1.0 g/L DS (black diamond). The error bars indicate standard deviations calculated from the data of three independent experiments.

Regardless of the concentration used, supplementation with DS or heparin did not significantly affect the growth of CHO-BMP-2 (Fig. 7a,b), but dramatically increased the rhBMP-2 production (Fig. 7c). DS was more effective than heparin in increasing the rhBMP-2 production. The maximum rhBMP-2 concentration, which was determined using ELISA, was achieved on day 6, and thereafter, decreased rapidly in all the conditions. The maximum rhBMP-2 concentrations at 1 g/L of DS (11.0 ± 0.7 μg/mL) and 0.2 g/L of heparin (9.5 ± 0.5 μg/mL) were 22.0- and 19.0-times higher than that of the control culture (0.5 ± 0.0 μg/mL; *p* < 0.05), respectively. The increased rhBMP‐2 production with supplementation with DS or heparin was also confirmed using western blot analysis (Supplementary Fig. S4). Supplementation with DS or heparin did not affect the mRNA level of rhBMP-2 during the exponential phase of growth (Supplementary Fig. S5). Thus, it is likely that supplementation with DS or heparin increased the apparent specific rhBMP-2 productivity (*q*_rhBMP‐2_) mainly by inhibiting HSPG-mediated internalization. Based on the data collected during the exponential growth phase, *q*_rhBMP‐2_ at 1 g/L DS (0.24 ± 0.01 μg/10^6^ cells/day) and 0.2 g/L heparin (0.20 ± 0.02 μg/10^6^ cells/day) were 12.0- and 10.0-times higher than that of the control culture (0.02 ± 0.00 μg/10^6^ cells/day; *p* < 0.05), respectively.

**Figure 8:**
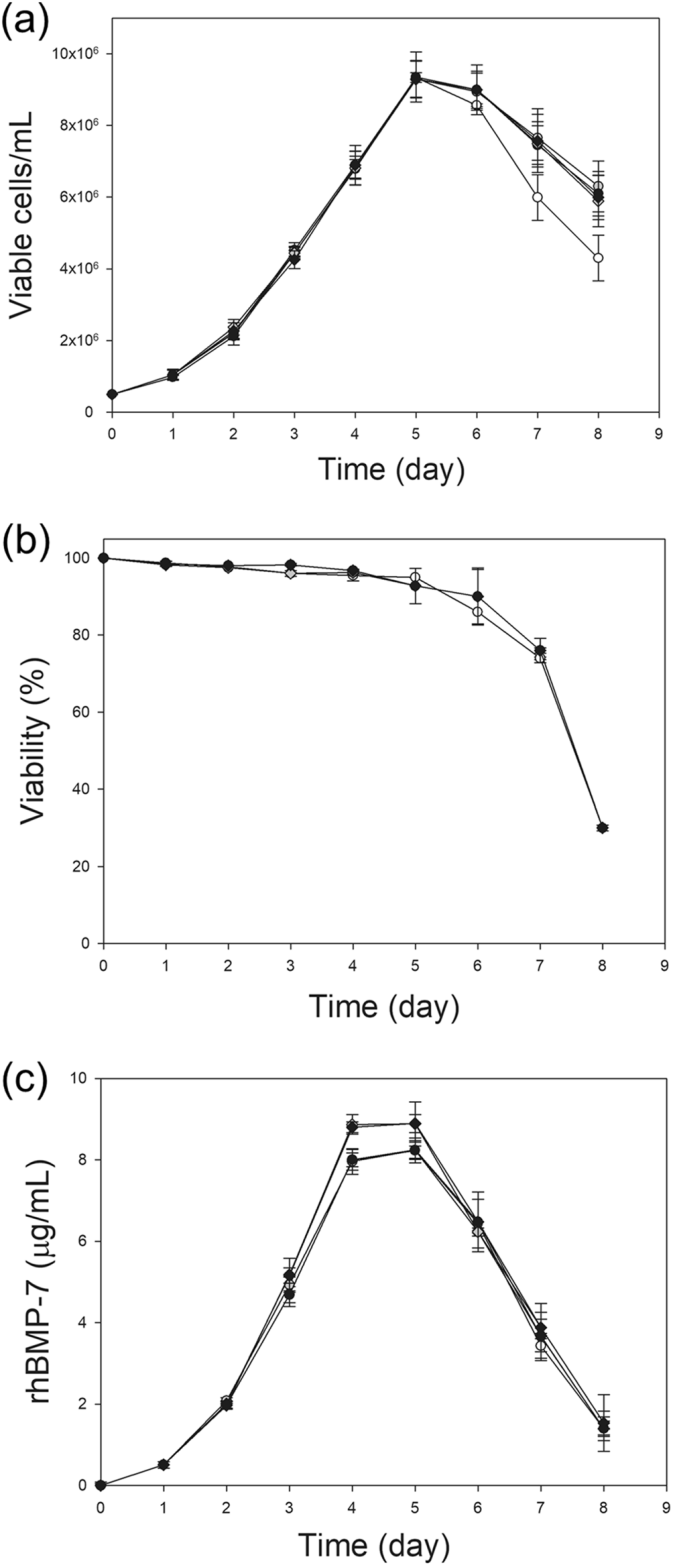
Supplementation of DS or heparin in CHO-BMP-7 cells cultures. Profiles of (**a**) cell growth, (**b**) viability, and (**c**) rhBMP-7 concentration during cultures supplemented with DS or heparin. No supplementation (white circle), supplementation with 0.1 g/L heparin (gray square), 0.2 g/L heparin (black square), 0.5 g/L DS (gray diamond), and 1.0 g/L DS (black diamond). The error bars indicate standard deviations calculated from the data of three independent experiments.

Similar to the effect seen in CHO-BMP-2 cells, growth of CHO-BMP-7 cells was also not significantly affected upon supplementation with DS or heparin (Fig. 8a,b). However, unlike rhBMP-2, no dramatic increase in rhBMP-7 production was observed upon supplementation with DS or heparin (Fig. 8c), which was also confirmed by western blot and qRT-PCR analyses (Supplementary Figs. S6, S7). The maximum rhBMP-7 concentration at 1 g/L of DS (8.8 ± 0.6 μg/mL) and 0.2 g/L of heparin (8.2 ± 0.2 μg/L) was slightly higher or similar to that of the control culture (8.2 ± 0.3 μg/mL). This result was expected because extracellular rhBMP-7 is not internalized into DG44 cells.

## Discussion

Approximately one-third of the TGF-β cytokine superfamily, including BMP-2 and BMP-7, are known to bind HS^20^. If the binding of secreted rhBMP-2 and rhBMP-7 to surface HSPGs leads to cellular internalization and endosomal degradation, the product yields of rhBMP-2 and rhBMP-7 in rCHO cell cultures could be improved by blocking the endocytosis of rhBMP-2 and rhBMP-7 into rCHO cells.

When rhBMP-2 or rhBMP-7 (10 μg/mL) in the culture medium was incubated with CHO cells, both rhBMP-2 and rhBMP-7 bound to cell surface HSPGs. However, only rhBMP-2 was internalized into CHO cells via cell surface HSPG-mediated endocytosis. After incubation with CHO cells for 24 h at 37 °C, the rhBMP-4 concentration in the culture medium decreased by approximately 75%. Active internalization of rhBMP-2 was confirmed using western blot analysis and confocal microscopy.

Cell surface HSPGs serve as a co-receptor for several HS-binding growth factors such as Dpp (a fly orthologue of BMPs) in Drosophila cells and FGF-2 in rat endothelial cells and HeLa cells, thus facilitating receptor-mediated endocytosis^7,22^. In contrast, cell surface HSPGs serve as the main internalizing receptors for FGF-2 and rhBMP-4 in CHO cells^8,23^, suggesting that the role of HSPGs depends on the cell line. In this study, cell surface HSPGs were found to act as the main internalizing receptor for rhBMP-2 in CHO cells. The cellular uptake of rhBMP-2 was not affected by the knockdown of both the BMP receptor genes (*bmpr1a* and *bmpr2*) and the BMP antagonist gene (*twsg1*). However, knockdown of the HSPG synthesis gene (*xylt2*) significantly decreased the cellular uptake of rhBMP-2. Furthermore, the internalized cell surface HSPGs and rhBMP-2 co-localized intracellularly.

Two major HSPG-mediated endocytosis pathways are clathrin- and caveolin-dependent pathways^24^. The choice of the endocytosis pathway depends on the type of cellular- and ligand^24–27^. The clathrin-dependent pathway is the major route for rhBMP-4 internalization in CHO cells^8^, whereas the caveolin-dependent pathway is the major route for rhFGF-2 internalization in BHK cells^28^. Both clathrin- and caveolin-dependent pathways were found to be the major routes for rhBMP-2 internalization in CHO and C2C12 cells^19^. Internalization of rhBMP-2 was significantly reduced in CHO cells pretreated with chlorpromazine and genistein, which inhibit the clathrin-dependent and caveolin-dependent pathways, respectively. Pretreatment of CHO cells with dynosore, which inhibits both the clathrin-dependent and caveolin-dependent pathways, also significantly reduced the rhBMP-2 internalization. Furthermore, direct evidence for HSPG-mediated endocytosis of rhBMP-2 via clathrin-dependent and caveolin-dependent pathways in CHO cells was provided using confocal microscopy that showed the co-localization of rhBMP-2 and HSPGs with clathrin or caveolin.

DS and heparin, which are linear anionic sulfated polysaccharides with similarity to HS chains, were successfully used to prevent the internalization of rhBMP-4 in CHO cells, resulting in a 1.4- and 1.1-fold increase in the maximum rhBMP-4 concentration in batch culture, respectively^8^. Similar to rhBMP-4, there was an increase in the maximum rhBMP-2 concentration upon addition of heparin or DS, but to a much higher extent. Addition of heparin and DS resulted in an 18.0–20.3-fold increase in the maximum rhBMP-2 concentration. The differential efficacy of the HS inhibitors between rhBMP-2 and rhBMP-4 is probably because rhBMP-2 was more rapidly internalized into CHO cells than rhBMP-4. When incubated at 37 °C with CHO cells, the concentration of rhBMP-2 decreased below 50% of the original concentration within 6 h. In contrast, the concentration of rhBMP-4 was approximately 50% of the original concentration after 24 h^8^. Unlike rhBMP-2, rhBMP-4 was internalized into CHO cells only through clathrin-mediated endocytosis, which was strongly inhibited during mitosis^29^. Thus, rhBMP-4 internalization is likely to be halted during cell division. A rapid decrease in rhBMP-2 concentration was also observed when incubated with C2C12 cells^30^.

Unlike rhBMP-2, rhBMP-7 was not internalized into CHO cells, although it binds to cell surface HSPGs. As expected, no dramatic increase in the maximum rhBMP-7 concentration was obtained by supplementation with DS or heparin. The requirements for cell surface HSPG-mediated endocytosis of ligands are not clearly understood, but the HS sulfation pattern of HSPGs is known to be one of the important factors that determine which ligand will be internalized by HSPGs. The HS side chains of HSPGs undergo extensive modification including N-deacetylation/sulfation and 6- and 3-O-sulfation of glucosamine, and 2-O-sulfation of iduronic acid^9^. These modification patterns, referred to as sulfation codes, contribute to the structural diversity of HS chains and enable cell surface HSPGs to interact with numerous proteins^9,31^. However, BMP-2 and BMP-7 recognize the same sulfation pattern^32,33^; therefore, other factors are likely to be involved in the selective endocytosis of rhBMP-2 into CHO cells.

It is believed that BMPs are attached to the cell surface HSPG via the spatial arrangement of basic amino acid stretches called Cardin and Weintraub (CW) structures^11,25^. BMP-2 possesses two major heparin-binding domains provided by two CW structures in the N-terminus of each monomer. In contrast, the BMP-7 dimer has a single heparin-binding domain originating from the two CW structures in the C-terminus of each monomer. The different numbers of heparin-binding domains in BMP-2 and BMP-7 may affect their binding avidity to HSPGs. In fact, the binding avidity between domain 1 of recombinant human perlecan, an extracellular matrix proteoglycan with three HS side chains linked to a large core protein, and rhBMP-2 was found to be 10- fold tighter than its binding avidity to rhBMP-7 in vitro^34^. Furthermore, the different locations of the heparin-binding domains may lead to the different spatial orientations of these BMPs with respect to the HS chain backbone. According to the mechanism of conventional receptor-mediated endocytosis, the binding avidity to receptors and the initial orientation of ligands are two major factors that determine which ligands are to be internalized^35,36^. Nevertheless, it is necessary to determine which of these factors are involved in HSPG-mediated endocytosis of rhBMPs.

In conclusion, both rhBMP-2 and rhBMP-7 in the culture medium bind to cell surface HSPGs in CHO cells. However, only rhBMP-2 is internalized into CHO cells through the clathrin- and caveolin-dependent pathways, and this internalization process is mainly regulated by HSPGs. Therefore, supplementation with DS or heparin, a competitive inhibitor of HSPGs, dramatically increased rhBMP-2 production by blocking the internalization of rhBMP-2 into rCHO cells. Thus, understanding the HSPG-mediated endocytosis of rhBMPs is necessary to improve rhBMP production in rCHO cell cultures.

## Methods

### Cell lines and culture maintenance

The rCHO cell lines that produce rhBMP-2 (CHO-BMP-2) and rhBMP-7 (CHO-BMP-7) were established from dihydrofolate reductase (dhfr)‐deficient CHO host cells (DG44) using a dhfr/methotrexate (MTX)-mediated gene amplification system and were adapted to grow in suspension, as described previously^5^. Suspension cultures were performed in a 125-mL Erlenmeyer flask (Corning, Corning, NY) on a Climo-Shaker (ISF1‐X, Adolf Kuhner AG, Birsfelden, Switzerland) at 110 rpm in a humidified 5%CO_2_/air mixture at 37 °C. The culture media used for CHO-BMP-2 and CHO-BMP-7 were PowerCHO (Lonza, Walkersville, MD) supplemented with 2 μM MTX and 8 mM glutamine and PowerCHO supplemented with 2 μM MTX (Sigma‐Aldrich, St. Louis, MO), 8 mM glutamine, and 300 μg/mL zeocin (Invitrogen, Carlsbad, CA), respectively. The DG44 cells were cultivated in Iscove's modified Dulbecco's medium (IMDM, Invitrogen) supplemented with 7% (v/v) dialyzed fetal bovine serum (Sigma‐Aldrich) and 1 × hypoxanthine/ thymidine (HT) (Gibco, Grand Island, NY).

### Incubation of purified rhBMP‐2 or rhBMP-7 with DG44 cells

Cells were plated at a concentration of 0.5 × 10^6^ cells/mL in a 24-well culture plate (Thermo Scientific, Rockford, IL) containing 1 mL of culture medium. After cultivation for 24 h, cells in the 24-well culture plate were washed with phosphate-buffered saline (PBS) and then incubated with 10 μg/mL of rhBMP‐2 or rhBMP-7 in IMDM plus 1 × HT at 4 °C and 37 °C. Cells were also incubated with 10 μg/mL of rhBMP‐2 or rhBMP-7 in IMDM plus 1 × HT in the presence of 0.5 g/L DS (MW 15,000 Da, Sigma-Aldrich) or 0.1 g/L heparin (Sigma-Aldrich) at 37 °C. Heparin and DS were prepared as stock solutions, as described previously^8^. For a cell-free control, 1 mL of IMDM plus 1 × HT containing 10 μg/mL of rhBMP‐2 or rhBMP-7 was also incubated at 4 °C and 37 °C. At the indicated time points, the supernatants were harvested and stored at − 70 °C for further analyses. For determination of rhBMP-2 and rhBMP-7 internalization, cells treated with heparinase I/III (Sigma-Aldrich; H3917) for selective removal of the HSPG chains were also prepared in a 24-well plate, as described previously^8^. Cells with or without heparinase treatment were incubated at 37 °C for 3 h with 10 μg/mL of rhBMP‐2 or rhBMP-7 in 1 mL of IMDM containing 1 × HT in the absence or presence of 0.5 g/L DS or 0.1 g/L heparin. The cells were then washed with ice-cold acidic buffer (0.5 M NaCl, 0.2 M acetic acid, pH 2.8) and PBS to remove the surface‐bound rhBMP. The cells were then detached from the 24-well culture plate by trypsinization and stored at − 70 °C for further analyses.

### Confocal microscopy

Cells were inoculated at a concentration of 2 × 10^4^ cells/well in a 96-well μ-plate (Ibidi, Munich, Germany) and grown for 24 h in a humidified 5% CO_2_/air mixture at 37 °C. For determination of rhBMP-2 and rhBMP-7 internalization, cells with or without heparinase treatment in a 96-well μ-plate were incubated in the conditions described earlier. To remove the surface‐bound rhBMP, cells were washed with ice-cold acidic buffer and PBS. The cells were then fixed with 4% paraformaldehyde, permeabilized using 0.1% Triton X-100 in PBS, and incubated with a blocking buffer (3% bovine serum albumin in PBS) at room temperature for 1 h. Cells incubated with rhBMP‐2 were stained with the primary antibodies, goat anti‐human BMP‐2 antibody (R&D systems, Minneapolis, MN; AF355) and mouse anti-HS antibody (Amsbio, Abingdon, UK; 370255) overnight at 4 °C, followed by secondary staining with Alexa Fluor 594 anti‐goat IgG (Abcam, Cambridge, UK; ab150132) and Alexa Flour 488 anti-mouse IgG (Thermo Scientific; A-11059) for 1 h at room temperature. For cells incubated with rhBMP‐7, a goat anti-human BMP-7 antibody (R&D Systems; MAB3541) was used as the primary antibody. Nuclei were stained using 4′,6-diamidino-2-phenylindole (DAPI) (Sigma‐Aldrich) for 30 min at room temperature. Cell samples were visualized using an LSM 780 confocal laser‐scanning microscope (Carl Zeiss, Jena, Germany) using 63 × planachromatic lens (Plan Apochromat 63 × /1.4 Oil DIC) with oil immersion and analyzed with Zen Blue software.

For determination of rhBMP-2 and rhBMP-7 binding to the cell surface HSPGs, cells with or without heparinase treatment were incubated at 4 °C for 1 h with 10 μg/mL of rhBMP‐2 or rhBMP-7 in IMDM containing 1 × HT in the absence or presence of 1 g/L DS or 0.2 g/L heparin. The cells were then washed with ice-cold PBS. As a negative control, cells without any rhBMPs bound on the cell surface HSPGs were also prepared using an additional acidic washing step. The cells were then fixed with 4% paraformaldehyde and incubated with a blocking buffer at room temperature for 1 h. Subsequent antibody staining and confocal microscopic visualization steps were performed in the same manner as described earlier.

### Transient RNA interference

siRNA duplexes against *bmpr1a*, *bmpr2*, *twsg1*, and *xylt2*, and a negative control were designed previously^8^. Transient transfection of the siRNA duplexes into exponentially growing cells was performed using Lipofectamine RNAiMAX reagent (Invitrogen), as described previously^8^.

### Endocytosis inhibitor treatment

Endocytosis inhibitors (chlorpromazine, genistein, and dynasore; all from Sigma‐Aldrich) were prepared as stock solutions in distilled water or dimethyl sulfoxide (DMSO), as described previously^8^. Exponentially growing DG44 cells were incubated at 37 °C for 30 min in IMDM plus 1 × HT containing 10 µM chlorpromazine, 200 µM genistein, or 100 µM dynasore. As a control, cells in IMDM plus 1 × HT without an endocytosis inhibitor and cells in IMDM plus 1 × HT with 20 µL DMSO were also incubated under the same conditions. The cells were then incubated at 37 °C for 3 h in IMDM plus 1 × HT with 10 µg/mL of rhBMP-2. Following this, cells were washed with ice-cold acidic buffer and PBS to remove the surface-bound rhBMP‐2 and harvested by trypsinization for further analyses.

### Cell cultures supplemented with DS or heparin

Exponentially growing cells (CHO‐BMP‐2 and CHO-BMP-7) were inoculated at a concentration of 0.5 × 10^6^ cells/mL into 125 mL Erlenmeyer flasks containing 30 mL of culture medium supplemented with heparin (0.1 or 0.2 g/L) or DS (1 or 2 g/L) and then incubated in a Climo‐Shaker incubator set at 110 rpm, 85% humidity, and 37 °C. Culture samples were collected daily. Viable cells were distinguished from dead cells using the Trypan Blue dye exclusion method.

### Quantitative real‐time PCR (qRT‐PCR) analysis

Total RNA was isolated using RiboEx (Geneall, Seoul, Korea) and cDNA was prepared using the High-Capacity cDNA Reverse Transcription Kit (Applied Biosystems, Foster City, CA) according to the manufacturer’s instructions. The primers for qRT‐PCR were designed previously^8^. The sequences of the designed primers are listed in Supplementary Table S1. qRT‐PCR was performed in triplicate as described previously^6^. The expression level of each gene was calculated using the ΔΔCt method with normalization to *actr5* and *gapdh*.

### Western blot analysis and quantification of rhBMP-2 and rhBMP-7

Western blot analysis was performed as described previously^13^. The PVDF-based blotting membranes were cut prior to hybridization using antibodies. The antibodies used for the analysis included anti-human BMP-2 antibody (R&D Systems; MAB3551), anti-human BMP-7 antibody (R&D Systems; MAB3541), and anti-β-actin antibody (Sigma-Aldrich; clone AC-74). rhBMP-2 and rhBMP-7 concentrations in the medium were measured using human BMP-2 and human BMP-7 DuoSet ELISA development kits (R&D Systems; DY355 and DY354), respectively. All western blot membranes with visible edges were included in Supplementary Figure S9.

### Statistical analysis

The values were expressed as mean ± standard deviation, n ≥ 3. Data were analyzed using a two-tailed Student’s *t*-test. Differences between the means were considered significant at *p* < 0.05.

## Supplementary Information

Supplementary Information

## Data Availability

All data generated or analyzed during this study are included in this published article (and its Supplementary Information files). The datasets generated during and/or analyzed during the current study are available from the corresponding author on reasonable request.
