# Supplementary material for: Selective endocytosis of recombinant human BMPs through cell surface heparan sulfate proteoglycans in CHO cells: BMP-2 and BMP-7
Source: Sci Rep. 2021 Feb 9;11:3378. doi: 10.1038/s41598-021-82955-1 (PMC7873082; doi:10.1038/s41598-021-82955-1)
Supplement: Supplementary file 1 — Supplementary Information [file 41598_2021_82955_MOESM1_ESM.pdf]

Supporting Information

Selective endocytosis of recombinant human BMPs through cell surface heparan sulfate proteoglycans in CHO cells: BMP-2 and BMP-7

Mi Gyeom Kim[1], Che Lin Kim[2], Young Sik Kim[3], Ju Woong Jang[3], Gyun Min Lee [1,2]*

[1] Department of Biological Sciences, KAIST, Daejeon, Republic of Korea

[2] The Novo Nordisk Foundation Center for Biosustainability, Technical University of Denmark, Lyngby, Denmark

[3] Institute of Biomaterial and Medical Engineering, Cellumed, Seoul, Republic of Korea

---

Correspondence

Gyun Min Lee, Department of Biological Sciences, KAIST, 291 Daehak-ro, Yuseong-gu, Daejeon 34141, Republic of Korea.

Email: gyunminlee@kaist.ac.kr

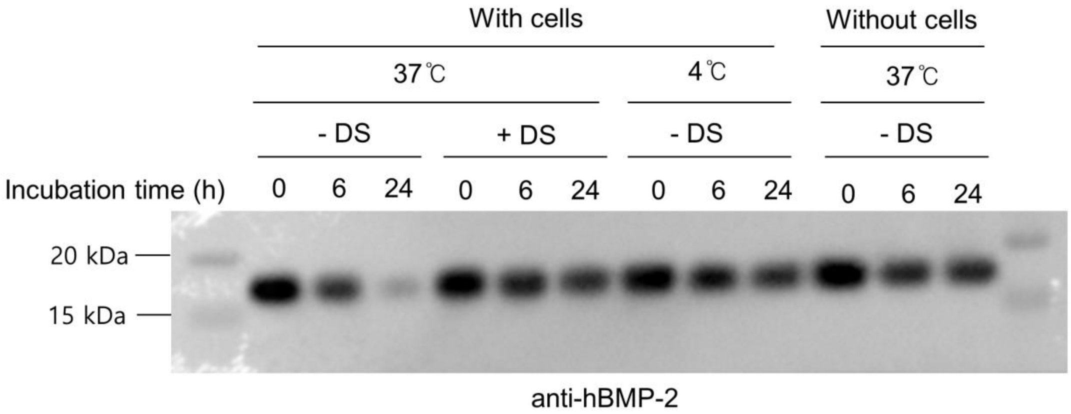

Supplementary Figure S1. Western blot analysis of medium containing 10 μg/mL of purified rhBMP-2 during incubation at 4°C or 37°C with and without cells in the presence (+) or absence (-) of DS (1 g/L) (refer to Figure 1a). An equal volume of the culture medium was separated on a 4–12% Bis–Tris NuPAGE gel under reducing conditions. The membrane was probed with an anti-human BMP-2 primary antibody (MAB3551, R&D systems), followed by an HRP-conjugated anti-mouse IgG secondary antibody (K0211589, Koma Biotech, Korea).

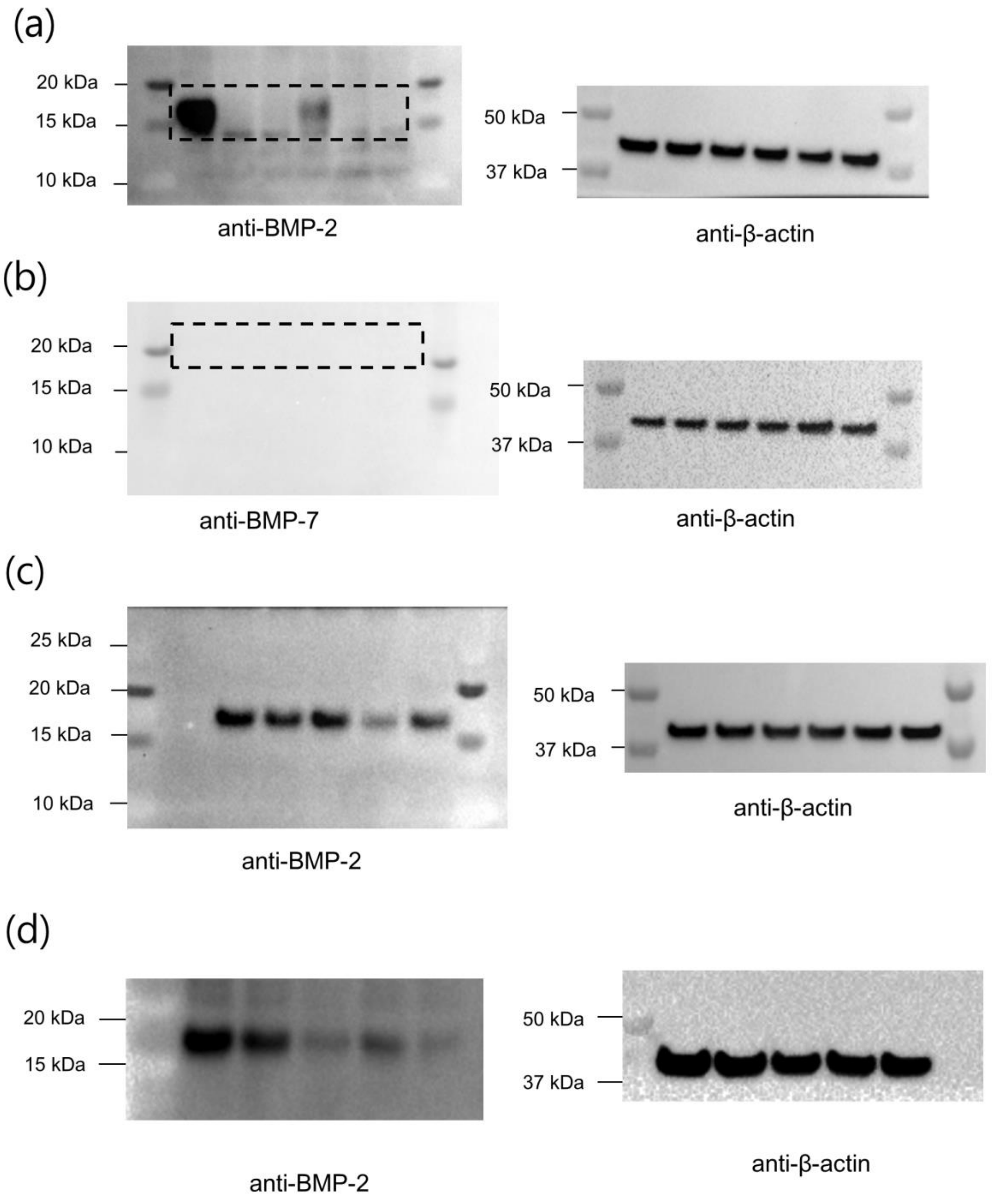

Supplementary Figure S2. Uncropped gel scans for all presented western blots. (a) corresponds to Fig 1b; (b) corresponds to Fig. 2b; (c) corresponds to Fig. 5b; (d) corresponds to Fig. 6.

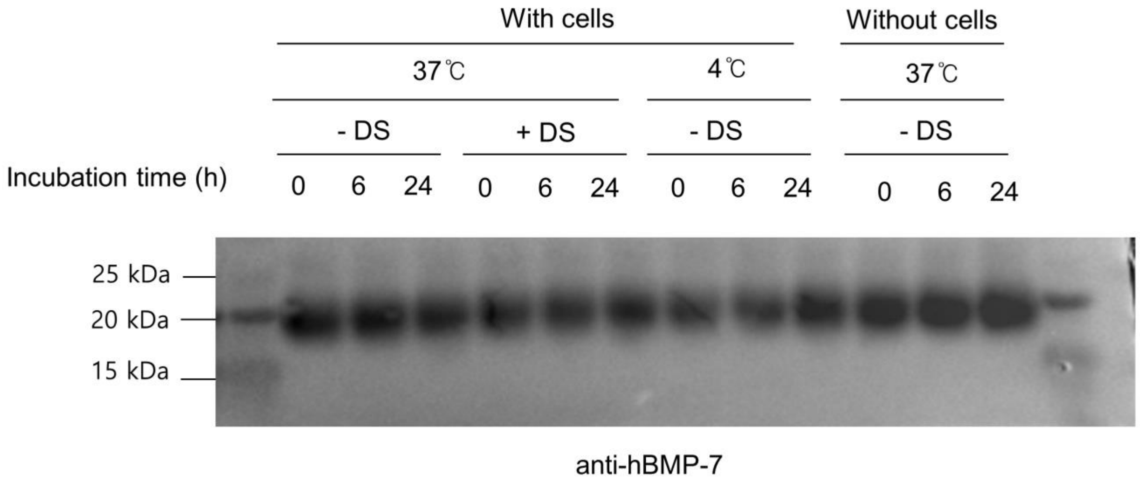

Supplementary Figure S3 Western blot analysis of medium containing 10 μg/mL of purified rhBMP-7 during incubation at 4°C or 37°C with and without cells in the presence (+) or absence (-) of DS (1g/L) (refer to Figure 2a). An equal volume of the culture medium was separated on a 4–12% Bis–Tris NuPAGE gel under reducing conditions. The membrane was probed with an anti-human BMP-7 primary antibody (MAB3451, R&D systems), followed by an HRP-conjugated anti-mouse IgG secondary antibody (K0211589, Koma Biotech, Korea).

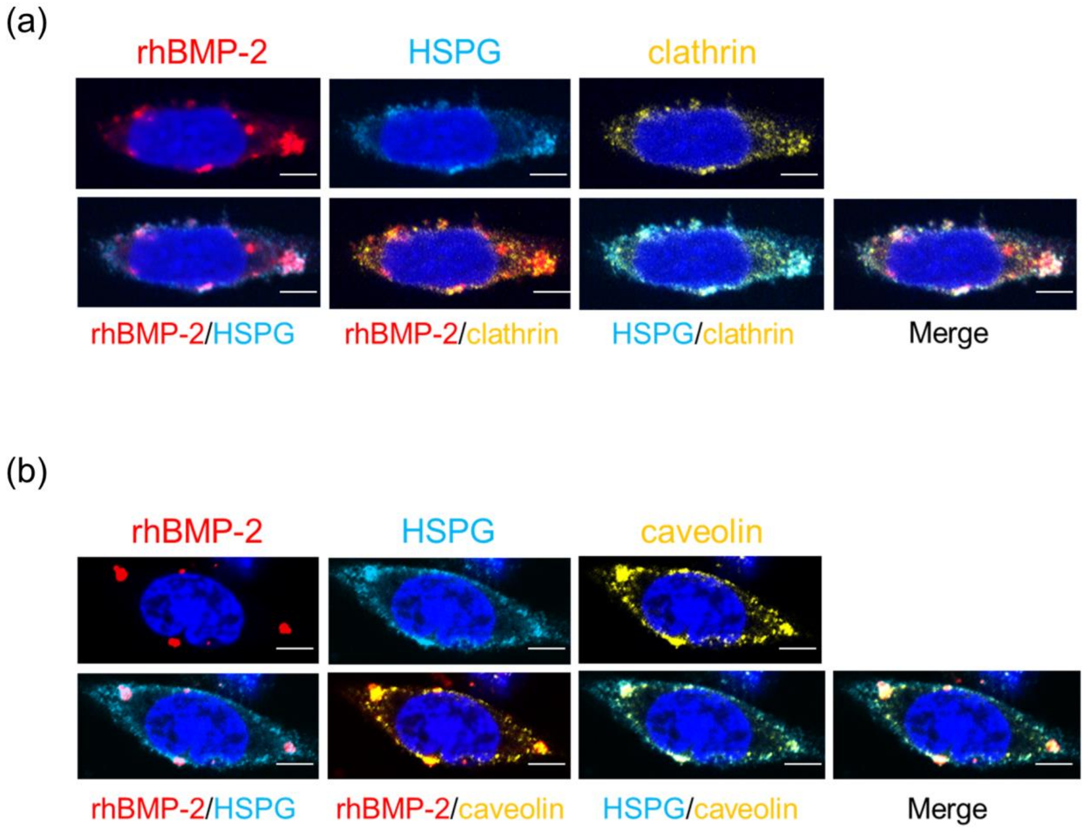

Supplementary Figure S4 Confocal microscopy to analyze the intracellular co-localization of cell surface HSPGs, rhBMP-2 and clathrin (a) or caveolin (b) in DG44 cells. Cells were incubated with 10 µg/mL of rhBMP-2 for 3 h at 37°C. After acidic washing to remove the surface-bound rhBMP-2, cells were fixed with 4% paraformaldehyde and permeabilized using 0.1% Triton X-100. After blocking, cells were incubated with anti-human BMP-2 antibody, anti-HS antibody, anti-clathrin antibody (ab21679, Abcam) or anti-caveolin antibody (ab2910, Abcam) overnight at 4°C. After washing with ice-cold PBS, cells were incubated for 1 h at room temperature with a mixture of secondary antibodies containing Alexa Fluor™ 488 donkey anti-mouse IgG H&L (A-21202, Thermo Scientific), Alexa Flour™ 568 donkey anti-goat IgG H&L (A-11057, Thermo Scientific), and Alexa Flour™ 647 donkey anti-rabbit IgG H&L (ab150075, Abcam), followed by nuclear staining with

DAPI. Scale bar, 5 μm.

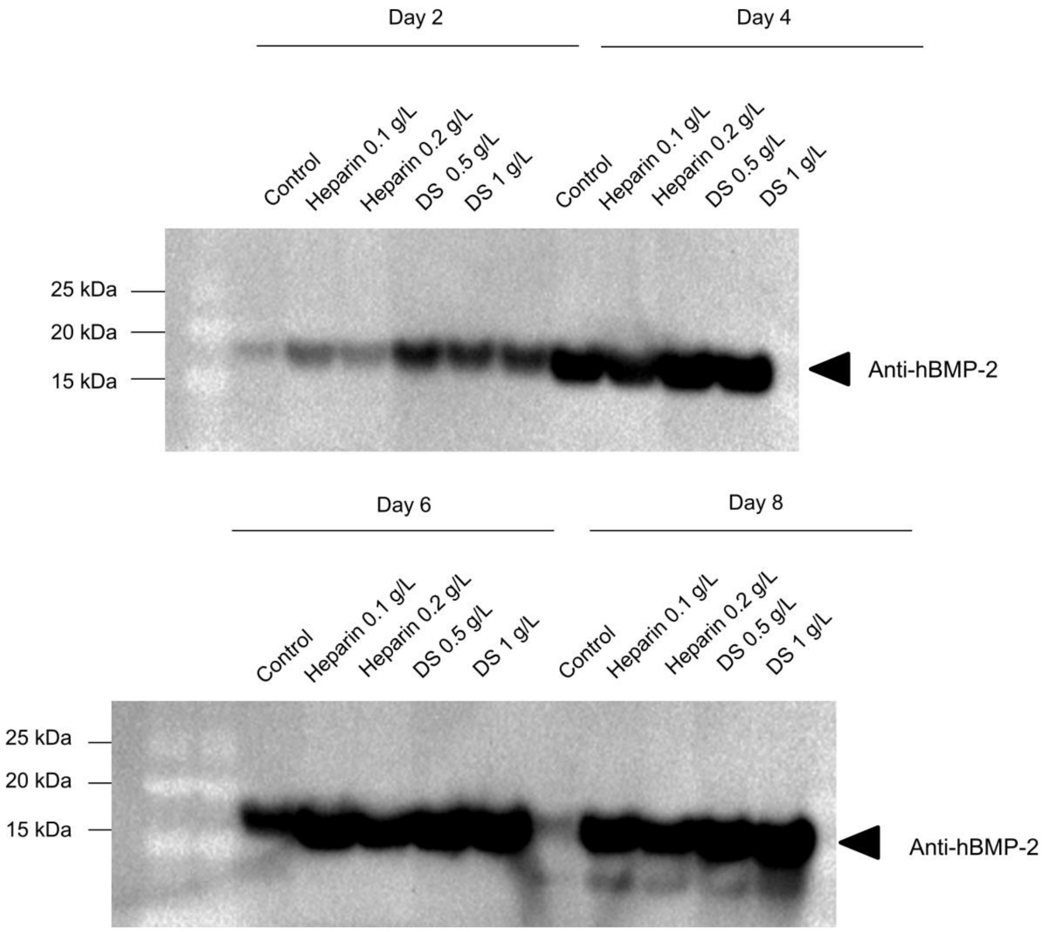

Supplementary Figure S5 Western blot analysis of culture supernatants in CHO-BMP-2 cell cultures in the absence or presence of DS (0.5 g/L or 1 g/L) or heparin (0.1 g/L or 0.2 g/L). Culture supernatant was sampled at indicated time points. An equal volume of the culture medium was separated on a 4–12% Bis–Tris NuPAGE gel under reducing conditions. The membrane was probed with an anti-human BMP-2 primary antibody (MAB3551, R&D systems), followed by an HRP-conjugated anti-mouse IgG secondary antibody (K0211589, Koma Biotech).

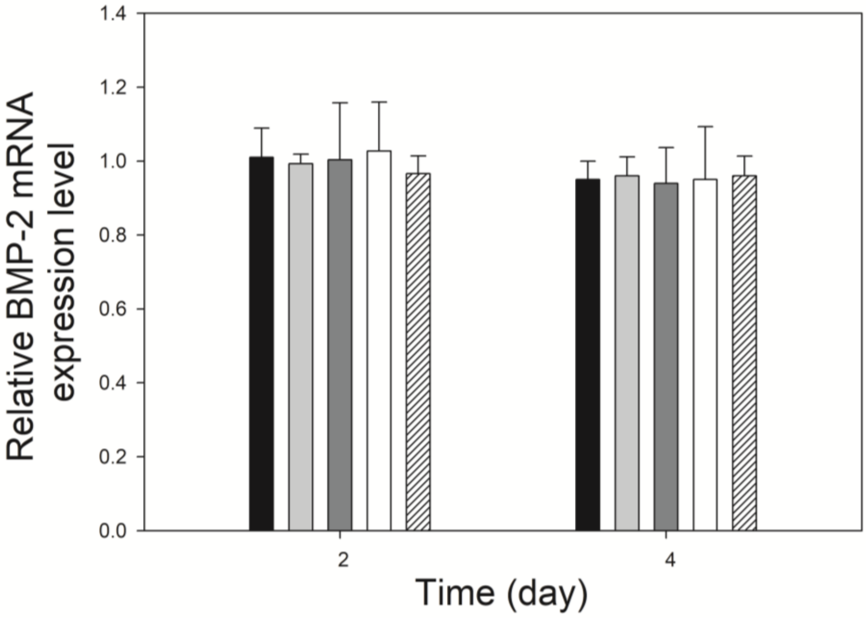

Supplementary Figure S6 Relative mRNA expression level of rhBMP-2 during cultures shown in Figure 7. Cells were sampled on days 2 and 4. Values were normalized to *gapdh* and then calculated based on the value of the control cell cultures without supplementation (black bar). 0.1 g/L heparin (light gray bar), 0.2 g/L heparin (dark gray bar), 0.5 g/L DS (white bar), and 1.0 g/L DS (dashed bar). The error bars indicate standard deviations calculated from the data of three independent experiments.

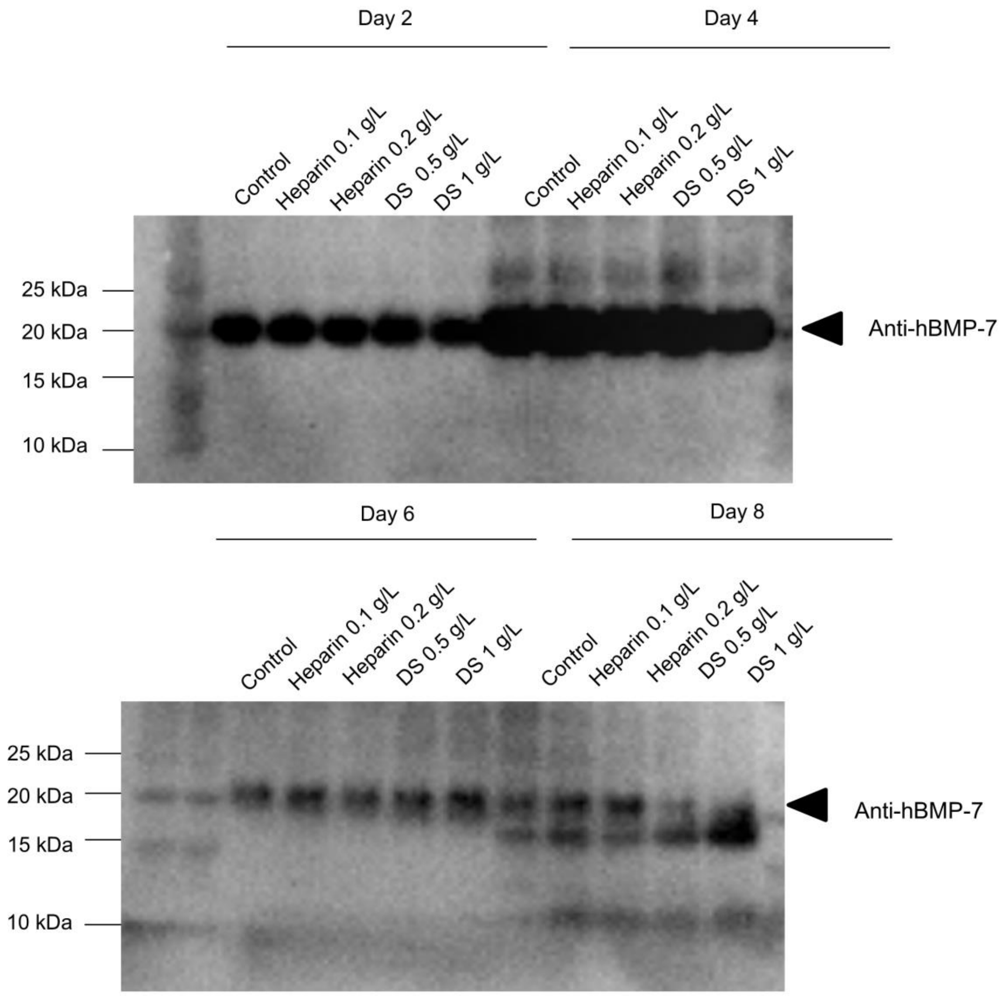

Supplementary Figure S7 Western blot analysis of culture supernatants in CHO-BMP-7 cell cultures in the absence or presence of DS (0.5 g/L or 1.0 g/L) or heparin (0.1 g/L or 0.2 g/L). Culture supernatant was sampled at indicated time points. An equal volume of the culture medium was separated on a 4–12% Bis–Tris NuPAGE gel under reducing conditions. The membrane was probed with anti-human BMP-7 primary antibody (MAB3541, R&D systems), followed by HRP-conjugated anti-mouse IgG (K0211589, Koma Biotech) as secondary antibody.

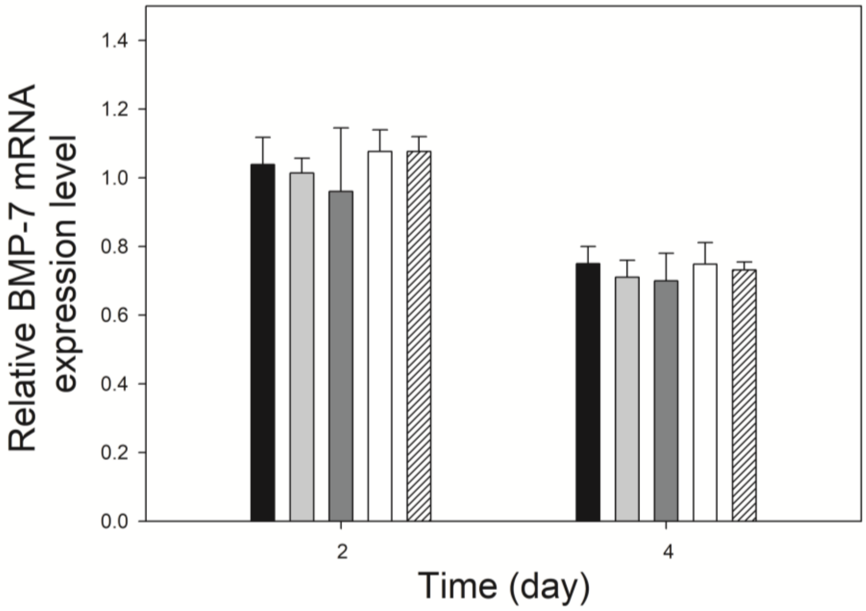

Supplementary Figure S8 Relative mRNA expression level of rhBMP-7 during cultures shown in Figure 8. Cells were sampled on days 2 and 4. Values were normalized to *gapdh* and then calculated based on the value of the control cell cultures without supplementation (black bar). 0.1 g/L heparin (light gray bar), 0.2 g/L heparin (dark gray bar), 0.5 g/L DS (white bar), and 1.0 g/L DS (dashed bar). The error bars indicate standard deviations calculated from the data of three independent experiments.

Gene name	Accession number	qRT-PCR primer set#1 (5'-3')*	qRT-PCR primer set#2 (5'-3')*
bmp2	XM_007615787.2	Forward:<br>GGGTGGAATGACTGGATTGT<br>Reverse:<br>GAGTTCAGATGATCAGCCAGAG	Forward:<br>CTACCAGAA ACGAGTGGGAAA<br>Reverse:<br>GAAGCTCTGCTGAGGTGATAAA
bmp7	XM_016971196.1	Forward:<br>CATCGAGAGTTCCGGTTTGA<br>Reverse:<br>TCCCGGATGTAGTCCTTGTA	Forward:<br>GCCGAATTCCGGATCTACAA<br>Reverse:<br>TCCTGGAGCACCTGATAAAC

Supplementary Table S1 Primer sequences used for qRT-PCR analysis.

* Primer set #1 and set #2 target different regions of each gene.

(a)

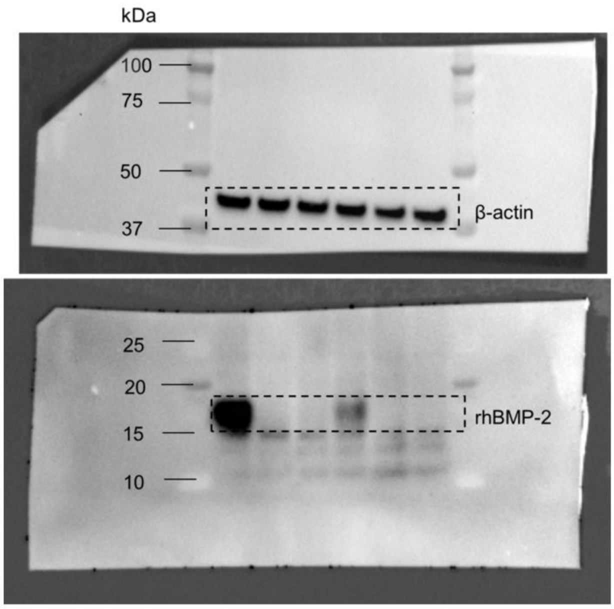
kDa
100
75
50
37
β-actin
25
20
15
10
rhBMP-2

(b)

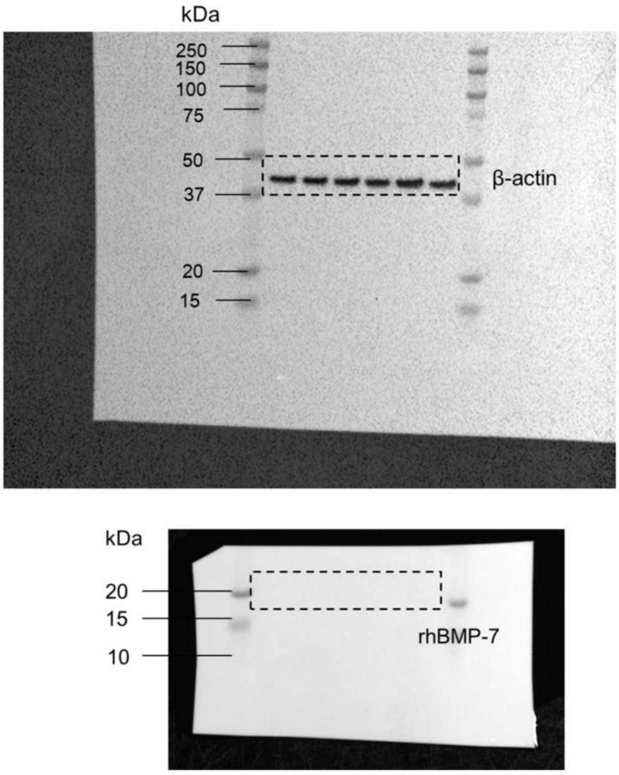
kDa
250
150
100
75
50
37
20
15
β-actin
kDa
20
15
10
rhBMP-7

(c)

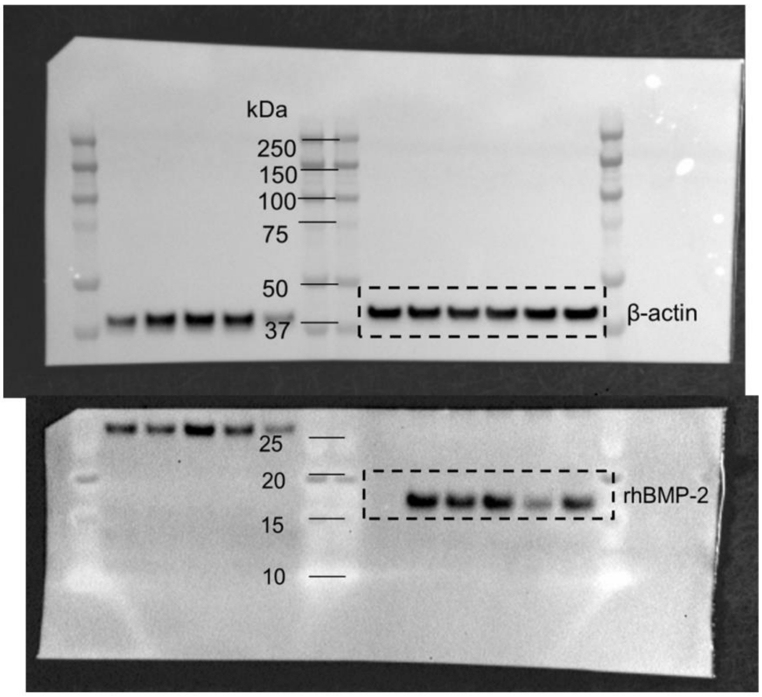
kDa
250
150
100
75
50
37
β-actin
25
20
15
10
rhBMP-2

(d)

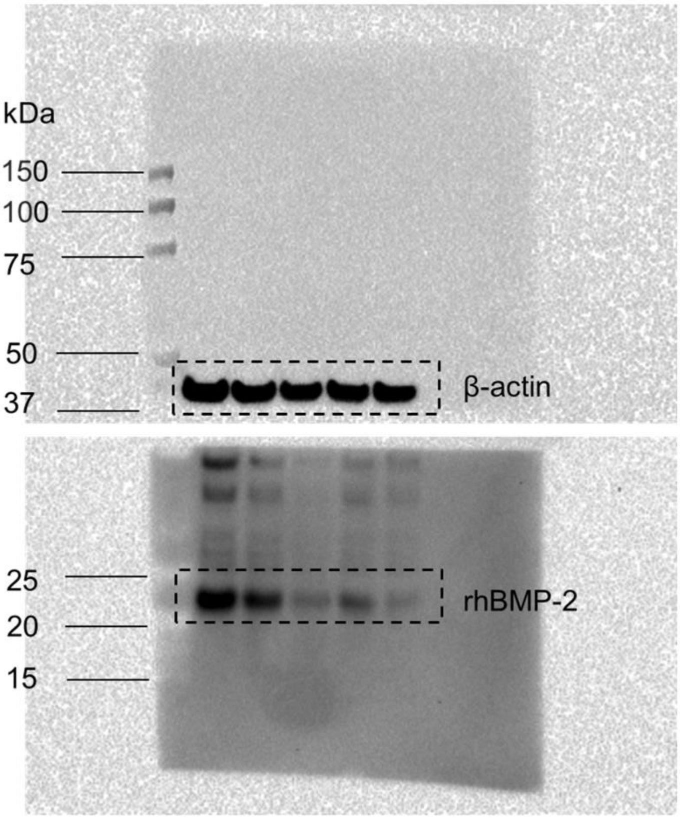
kDa
150
100
75
50
37
β-actin
25
20
15
rhBMP-2

(e)

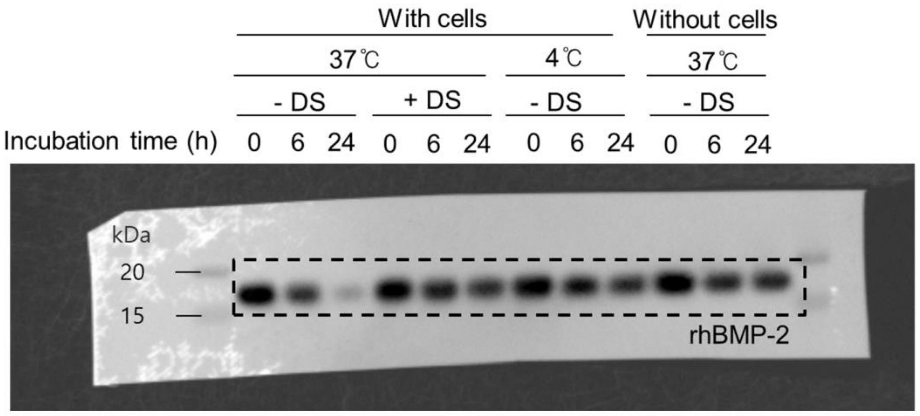
With cells
Without cells
37℃
4℃
37℃
- DS
+ DS
- DS
- DS
Incubation time (h)
0 6 24 0 6 24 0 6 24 0 6 24
kDa
20
15
rhBMP-2

(f)

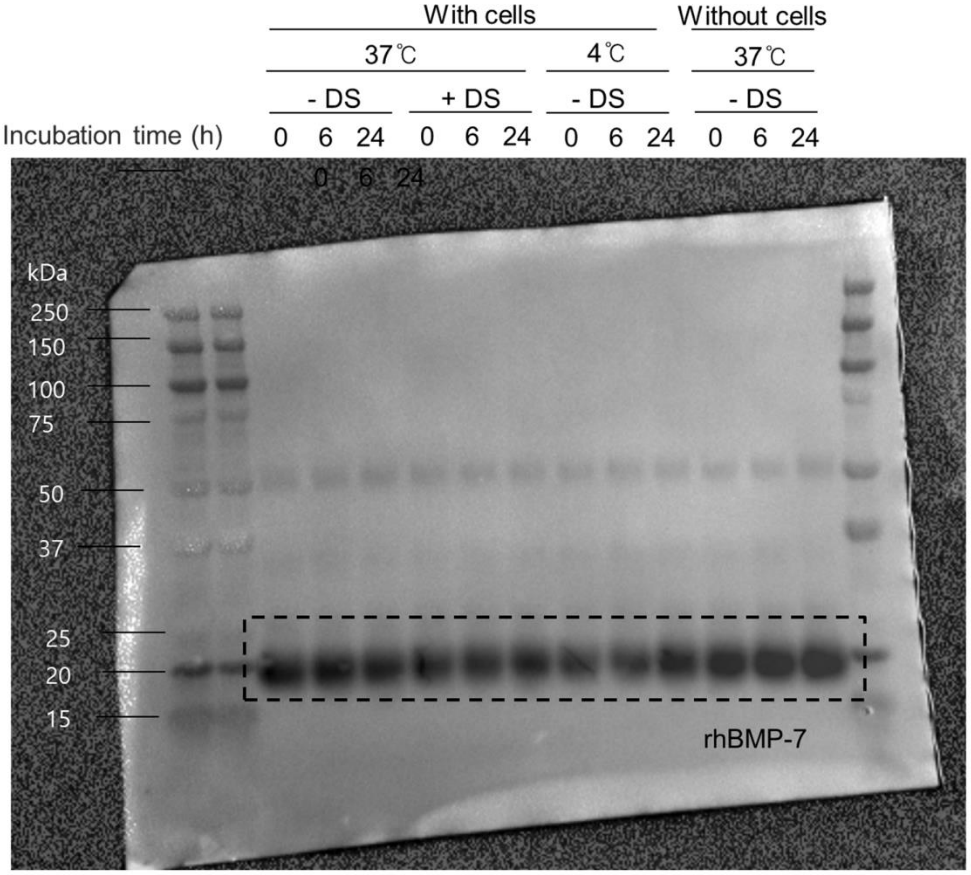
With cells
Without cells
37℃
4℃
37℃
- DS
+ DS
- DS
- DS
Incubation time (h)
0 6 24 0 6 24 0 6 24 0 6 24
0 6 24
kDa
250
150
100
75
50
37
25
20
15
rhBMP-7

(g)

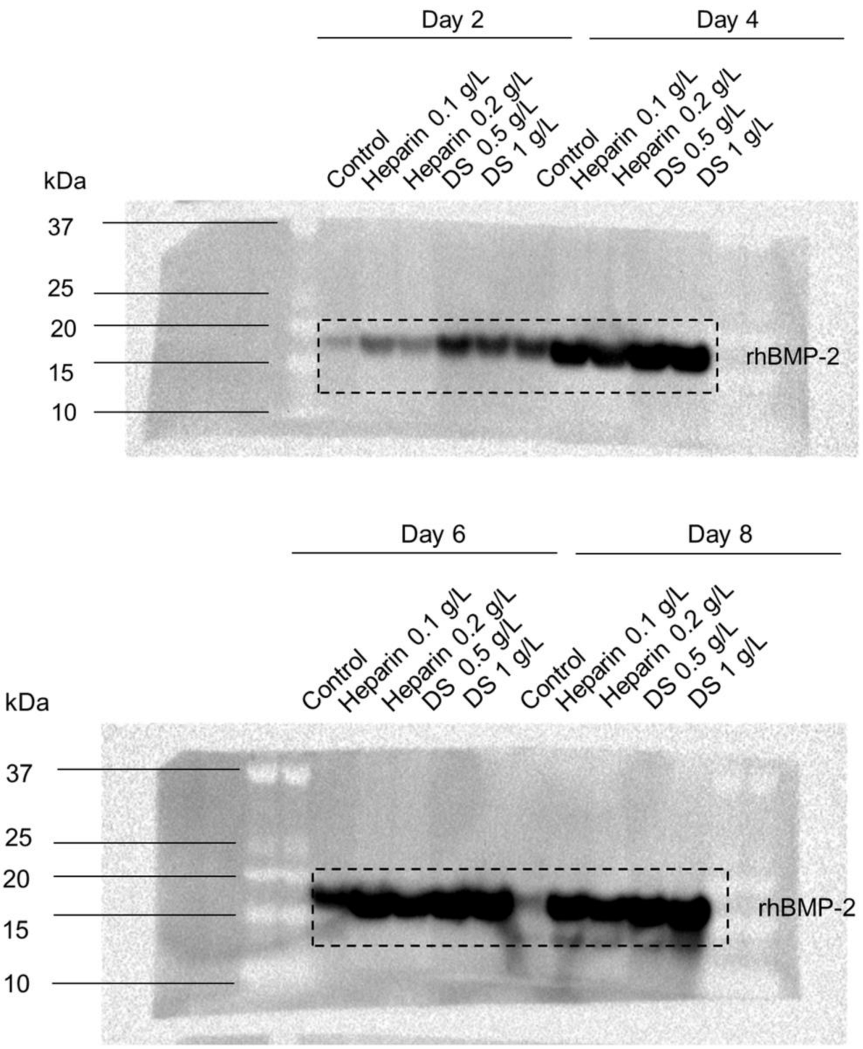
Day 2
Day 4
Control
Heparin 0.1 g/L
Heparin 0.2 g/L
DS 0.5 g/L
DS 1 g/L
Control
Heparin 0.1 g/L
Heparin 0.2 g/L
DS 0.5 g/L
DS 1 g/L
kDa
37
25
20
15
10
rhBMP-2
Day 6
Day 8
Control
Heparin 0.1 g/L
Heparin 0.2 g/L
DS 0.5 g/L
DS 1 g/L
Control
Heparin 0.1 g/L
Heparin 0.2 g/L
DS 0.5 g/L
DS 1 g/L
kDa
37
25
20
15
10
rhBMP-2

(h)

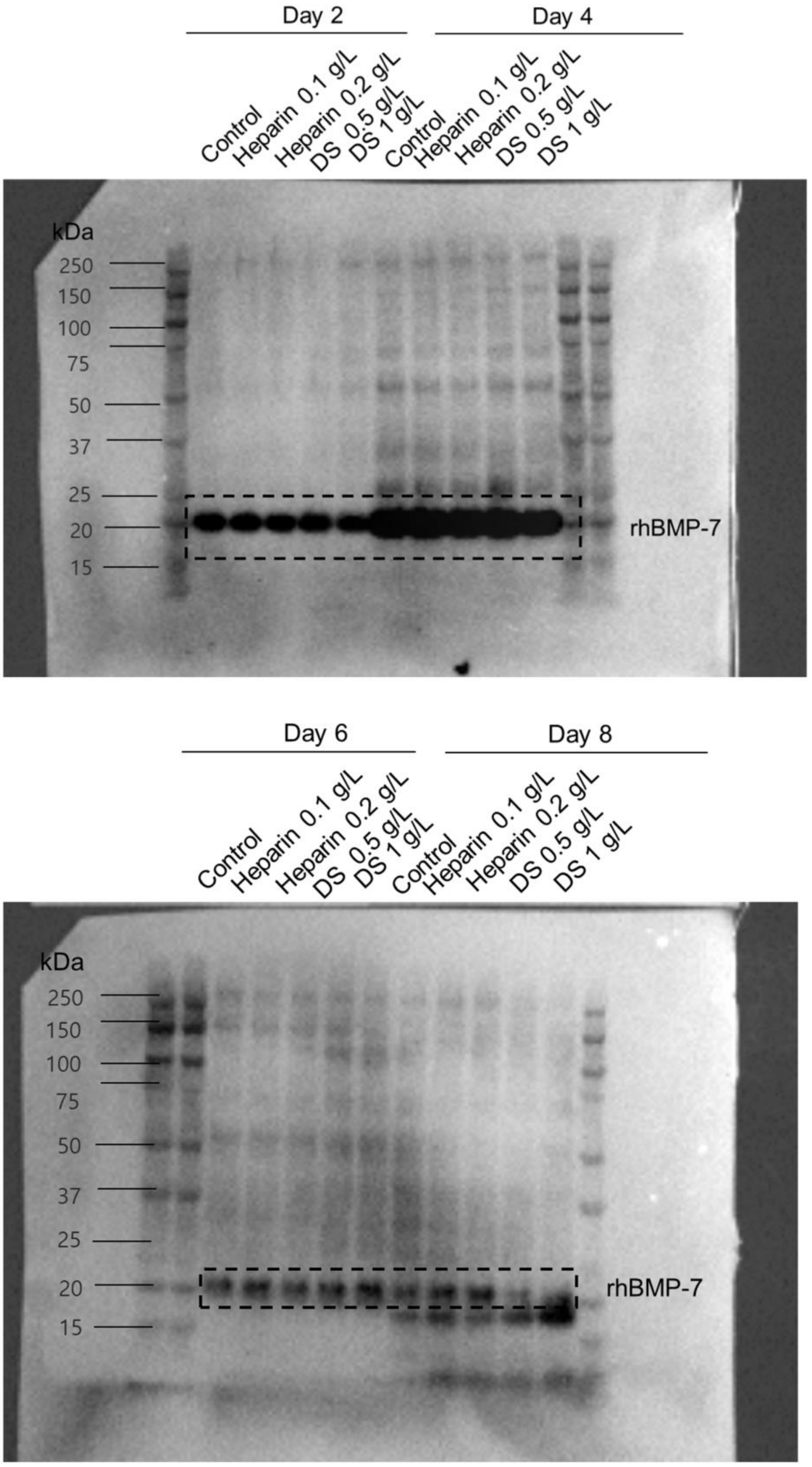
Day 2
Day 4
Control
Heparin 0.1 g/L
Heparin 0.2 g/L
DS 0.5 g/L
DS 1 g/L
Control
Heparin 0.1 g/L
Heparin 0.2 g/L
DS 0.5 g/L
DS 1 g/L
kDa
250
150
100
75
50
37
25
20
15
rhBMP-7
Day 6
Day 8
Control
Heparin 0.1 g/L
Heparin 0.2 g/L
DS 0.5 g/L
DS 1 g/L
Control
Heparin 0.1 g/L
Heparin 0.2 g/L
DS 0.5 g/L
DS 1 g/L
kDa
250
150
100
75
50
37
25
20
15
rhBMP-7

Supplementary Figure S9. All western blot membranes with visible edges. Most of the membranes were cut prior to hybridization using antibodies. (a) corresponds to Fig 1b; (b) corresponds to Fig. 2b; (c) corresponds to Fig. 5b; (d) corresponds to Fig. 6; (e) corresponds to Supplementary Figure S1; (f) corresponds to Supplementary Figure S3; (g) corresponds to Supplementary Figure S5; (h) corresponds to Supplementary Figure S7.
